# The scaffold protein XRCC1 stabilizes the formation of polβ/gap DNA and ligase IIIα/nick DNA complexes in base excision repair

**DOI:** 10.1016/j.jbc.2021.101025

**Published:** 2021-07-30

**Authors:** Qun Tang, Melike Çağlayan

**Affiliations:** Department of Biochemistry and Molecular Biology, University of Florida, Gainesville, Florida, USA

**Keywords:** base excision repair, DNA polymerase β, DNA ligase IIIα, X-ray cross-complementing protein 1, 5′-dRP, 5′-deoxyribose phosphate, AP, apurinic/apyrimidinic, APE1, AP endonuclease 1, APTX, aprataxin, BER, base excision repair, BLI, BioLayer Interferometry, CHO, Chinese hamster ovary, IPTG, isopropyl β-D-thiogalactoside, LB, Lysogeny Broth, NTD, N-terminal domain, OGG1, 8-oxoguanine DNA glycosylase, SEC, size-exclusion chromatography, SN-BER, single-nucleotide BER, SNP, single-nucleotide polymorphism, Tdp1, tyrosyl-DNA phosphodiesterase 1, XRCC1, X-ray cross-complementing protein 1

## Abstract

The base excision repair (BER) pathway involves gap filling by DNA polymerase (pol) β and subsequent nick sealing by ligase IIIα. X-ray cross-complementing protein 1 (XRCC1), a nonenzymatic scaffold protein, assembles multiprotein complexes, although the mechanism by which XRCC1 orchestrates the final steps of coordinated BER remains incompletely defined. Here, using a combination of biochemical and biophysical approaches, we revealed that the polβ/XRCC1 complex increases the processivity of BER reactions after correct nucleotide insertion into gaps in DNA and enhances the handoff of nicked repair products to the final ligation step. Moreover, the mutagenic ligation of nicked repair intermediate following polβ 8-oxodGTP insertion is enhanced in the presence of XRCC1. Our results demonstrated a stabilizing effect of XRCC1 on the formation of polβ/dNTP/gap DNA and ligase IIIα/ATP/nick DNA catalytic ternary complexes. Real-time monitoring of protein–protein interactions and DNA-binding kinetics showed stronger binding of XRCC1 to polβ than to ligase IIIα or aprataxin, and higher affinity for nick DNA with undamaged or damaged ends than for one nucleotide gap repair intermediate. Finally, we demonstrated slight differences in stable polβ/XRCC1 complex formation, polβ and ligase IIIα protein interaction kinetics, and handoff process as a result of cancer-associated (P161L, R194W, R280H, R399Q, Y576S) and cerebellar ataxia-related (K431N) XRCC1 variants. Overall, our findings provide novel insights into the coordinating role of XRCC1 and the effect of its disease-associated variants on substrate-product channeling in multiprotein/DNA complexes for efficient BER.

Base excision repair (BER) is a critical process for preventing the mutagenic and lethal consequences of DNA lesions such as apurinic/apyrimidinic (AP) sites and DNA base modifications arising from exposure to environmental hazards and various endogenous stressors (1, 2, 3, 4). If not repaired, they can lead to mutations or genomic instability, interfere with DNA replication or transcription, and the consequences can promote human diseases such as cancer and neurodegenerative disorders (5). One of the subpathways of BER, known as single-nucleotide or short-patch BER (SN-BER), requires the coordinated action of four core enzymes: DNA glycosylase, AP endonuclease 1 (APE1), DNA polymerase (pol) β, and DNA ligase IIIα (ligase IIIα) (6, 7, 8). The SN-BER pathway involves a series of sequential enzymatic steps that are tightly coordinated through protein–protein and protein–DNA interactions in a process referred to as “passing-the-baton” (9, 10, 11, 12). In this process, DNA substrates and reaction products are channeled from one step to the next in a processive fashion so that release of cytotoxic repair intermediates is minimized (13, 14, 15). At the initial step, many DNA glycosylases bind to the AP site product with higher affinity than the initial base damage substrate, implying that these proteins have evolved to protect cells from the adverse effects of AP sites and facilitate repair by signaling the next enzyme in the pathway (16). APE1 that cleaves the phosphodiester backbone leaving 3′-hydroxyl (3′-OH) and 5′-deoxyribose phosphate (5′-dRP) groups, likewise, exhibits higher affinity for its incised single-strand break product, coordinating with polβ (17). Polβ then binds to one nucleotide gap DNA and removes the 5′-dRP group and catalyzes template-directed gap filling DNA synthesis (18). The resulting nicked repair intermediate of polβ nucleotide insertion product is subsequently sealed during the final step of the BER pathway by ligase IIIα that catalyzes a phosphodiester bond formation between 3′-OH and 5′-phosphate (5′-P) ends (19, 20). Although biochemical studies and structural analyses with repair protein/DNA intermediate binary or ternary complexes extensively established the roles and activities of individual BER enzymes, how the repair enzymes function together in a multiprotein/DNA complex to facilitate the channeling of DNA intermediates in the coordinated repair pathway is poorly understood (21, 22). The scaffolding proteins play a key role in assembling sets of enzymes to perform multistep repair process, reducing the likelihood that labile repair intermediates are released leading to genome instability (23).

X-ray cross-complementing protein 1 (XRCC1) is a nonenzymatic protein known to be critical repair factor for coordinating BER (24, 25). XRCC1 protein is composed of N-terminal DNA-binding (NTD), central (BRCA1 C Terminus) BRCT-I, and C-terminal BRCT-II domains (26, 27). Current evidence indicates that the role of XRCC1 in the BER pathway is as a scaffolding factor mainly through its protein–protein and protein–DNA interactions to modulate the coordinated repair (28). For example, XRCC1 is recruited to DNA through its interaction with enzymes that recognize and bind specific lesions in the genome, such as has been reported for APE1 (29, 30, 31). XRCC1 also tightly interacts with polβ through its NTD, which is required for recruitment of the repair complex at the DNA damage site (32, 33, 34, 35, 36, 37). Many examples of protein–protein interactions for factors involved in the BER pathway have been reported, and in some cases, the interactions were found to impart a change in the activity of a BER enzyme (29, 30, 31, 32, 33, 34, 35, 36, 37). For example, the stimulating role of XRCC1 on the enzymatic activities of BER enzymes has been shown for APE1, PNK, 8-oxoguanine DNA glycosylase (OGG1), and Tyrosyl-DNA phosphodiesterase 1 (Tdp1) (30, 31, 38, 39). Additionally, XRCC1 forms a repair complex with ligase IIIα *via* its BRCT-II domain and operates to stabilize the enzyme intracellularly (20, 40, 41, 42, 43). XRCC1 also plays a role in the processing of the abnormal strand break ends for the continuity of the phosphodiester backbone through its interaction with the DNA-end processing enzyme Aprataxin (APTX) (44, 45). APTX removes an adenylate (AMP) from the 5′-end of ligation failure products and its deficiency is linked to the neurodegenerative ataxia disorder Ataxia-oculomotor apraxia 1 (AOA1) (46, 47, 48). It has been reported that a deficiency of XRCC1 leads to a reduction in APTX accumulation at the sites of DNA damage, and furthermore, XRCC1 mutations have been found to be associated with cerebellar ataxia, ocular motor apraxia, and axonal neuropathy (49). For example, the patient with cerebellar ataxia carrying XRCC1 K431N mutation combines phenotypic features of AOA1 and the cells exhibit dramatically reduced repair rates in response to oxidative DNA damage (50).

The biological importance of XRCC1 has been well established, dating back to early studies that demonstrated that Chinese hamster ovary (CHO) cells deficient in XRCC1 exhibit increased sensitivity to DNA alkylating agents, a higher level of DNA strand breaks, and genomic instability in the form of elevated sister chromatid exchanges (51, 52, 53, 54, 55). The biological importance of XRCC1 has also been suggested by the embryonic lethality of XRCC1 gene deletion, and studies of mouse embryonic fibroblast (MEF) cells isolated from early embryos exhibited hypersensitivity to DNA damage agents (56, 57, 58). Furthermore, the mice engineered for XRCC1 germline deletion exhibit a phenotype of embryonic lethality (53, 54, 55). Finally, single-nucleotide polymorphisms (SNPs) of XRCC1 have been found as risk factors for the development of different types of cancer (59, 60, 61, 62, 63). A reduced BER capacity and cellular transformation have been reported in cells expressing XRCC1 cancer-associated variants in response to DNA damaging agents (64, 65, 66). For example, the studies have reported the disparate patterns for the localization of XRCC1 and its interacting partners to the sites of DNA damage and altered repair profiles of oxidative damage induced H_2_O_2_ in the XRCC1-deficient EM9 CHO cells expressing XRCC1 cancer-associated variants R194W, R280H, and R399Q (59, 64).

Although the significance of XRCC1 in the maintenance of genome integrity and cellular functionality has been well established, it remains less clearly defined at the biochemical level that how XRCC1 coordinates the BER steps through its scaffolding function during the substrate-product channeling process particularly at the downstream steps of BER pathway. Similarly, even though the significance of XRCC1 variants in the cellular functionality has been well defined, their biochemical characterization in the BER regulation through the coordinated interactions with the key repair enzymes polβ, ligase IIIα, APTX that play critical roles at the final steps of the coordinated repair is poorly understood.

In the present study, we examined the role of XRCC1 on the efficiency of the substrate-product channeling process in reconstituted BER reactions *in vitro*. Furthermore, we characterized XRCC1 with its BER protein partners (polβ, ligase IIIα, and APTX) to investigate XRCC1-mediated repair protein complex formation, protein–protein interactions, and DNA-binding affinity to the repair intermediates including gap and nick DNA with or without damaged ends. For this purpose, we studied the wild-type, the cancer-associated variants (P161L, R194W, R280H, R399Q, Y576S) and the cerebellar ataxia-related (K431N) mutant of XRCC1 (Scheme S1). Our results revealed the stable polβ/XRCC1 repair protein complex formation through size-exclusion chromatography (SEC) and the stabilizing effect of XRCC1 on the formation of the catalytic ternary complexes polβ/dNTP/gap DNA and ligase IIIα/ATP/nick DNA. The real-time protein–protein interaction kinetics of XRCC1 showed little or no effect on the equilibrium binding constants (K*D*) between wild-type and variants while there were significant differences depending on the interacting repair protein partner of XRCC1 (polβ > ligase IIIα >> APTX). We observed higher binding affinity of XRCC1 for nick repair intermediate with preinserted 3′-dG:C or 3′-8-oxodG:A than one nucleotide gap DNA. Moreover, our results revealed a processivity role of XRCC1 in stimulating the channeling of repair products after polβ dGTP insertion opposite C in a gap and an enhanced ligation by ligase IIIα in complex with XRCC1, which is mediated through BRCT domains of both proteins. The mutagenic ligation of nick repair product with 8-oxodGMP inserted by polβ is also enhanced in the presence of XRCC1. Overall results could provide an insight into the mechanism by which XRCC1 orchestrates the passing-the-baton process particularly at the downstream steps of BER pathway and contribute to the understanding of how a multiprotein/DNA repair complex (polβ, ligase IIIα, APTX) is coordinated through the molecular interactions mediated by XRCC1.

## Results

### XRCC1 variants form stable protein complexes with polβ

We first investigated the repair protein complex formation and then validated the protein–protein binding affinities between polβ and XRCC1 through the SEC and GST-pull down analyses, respectively. For this purpose, we studied the wild-type XRCC1, polβ interaction mutants (V86R, R109A), the cancer-associated (P161L, R194W, R280H, R399Q, Y576S), and the cerebellar ataxia-related (K431N) variants of XRCC1 (Scheme S1).

**Figure 1:**
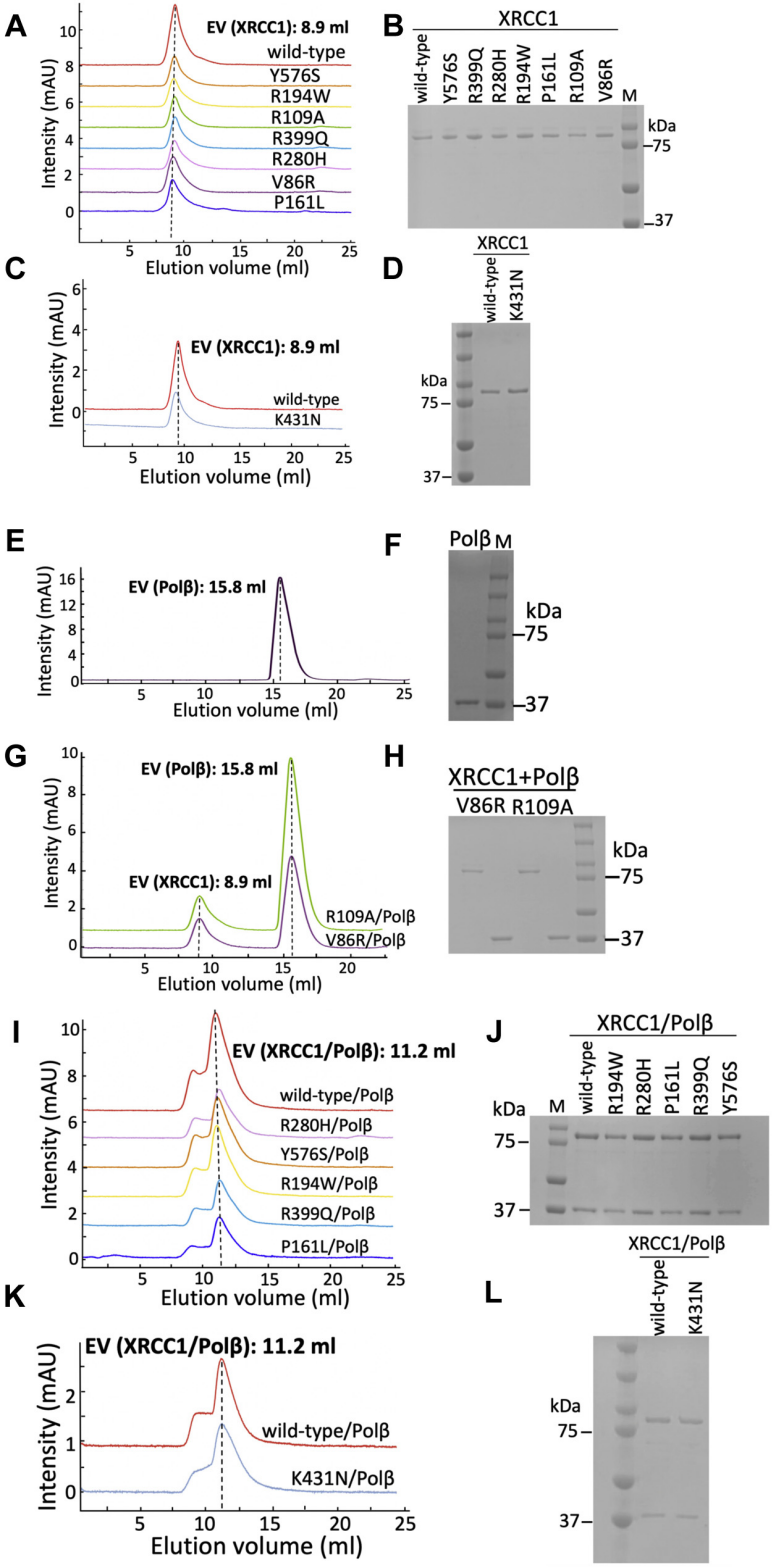
**Repair protein complex formation of XRCC1 and polβ.** Size-exclusion chromatography profiles showing the elution volumes (EV) for individual XRCC1 (*A*–*D*) and polβ (*E* and *F*) proteins are presented as in 8.9 and 15.8 ml, respectively, on the side of respective elution peaks. XRCC1/polβ interaction mutants V86R and R109A are eluted separately (*G* and *H*). XRCC1/polβ protein complexes are presented as EV: 11.2 ml for XRCC1 cancer-associated variants (*I* and *J*) and the cerebellar ataxia-related K431N mutant (*K* and *L*). Each peak fraction is analyzed on 12% SDS-polyacrylamide (w/v) gel and compared with the molecular weight marker (M: Precision Plus Protein Dual Color Standards, 10–250 kDa).

The elution peaks for the individual proteins of XRCC1 (wild-type and mutants) were obtained at 8.9 ml (Fig. 1, *A*–*D*). Polβ protein was eluted at 15.8 ml (Fig. 1, *E* and *F*). In line with the previously reported studies that demonstrate the destabilized interaction between polβ and XRCC1 carrying V to R substitution at position 86 (V86R), we showed that XRCC1 V86R mutant failed to form a complex with polβ as both proteins eluted separately with no shift evident in their individual elution volumes (Fig. 1, *G* and *H*). Furthermore, R to A substitution at position 109 (R109A) in the DNA-binding interface of XRCC1 has been reported as polβ interaction mutant in chemical shift mapping and structural prediction studies (34, 35, 36). Our SEC analysis of XRCC1 R109A mutant also showed no protein complex formation with polβ (Fig. 1, *G* and *H*). We obtained the protein complex of XRCC1/polβ, which was coeluted at 11.2 ml when these two proteins were mixed together (Fig. 1, *I*–*L*). Our results showed no difference in the protein complex elution shifts between XRCC1 wild-type and disease-related variants (Fig. 1, *I*–*L*). Furthermore, we confirmed the XRCC1/polβ protein complex formation *via* SEC analysis with the N-terminal domain (NTD) of the protein that is known to mediate XRCC1 interaction with polβ (Fig. S1). The elution peak position of XRCC1 NTD was at 17 ml, while the polβ/XRCC1 NTD protein complex was eluted at 14.4 ml. Overall results demonstrated a stable protein complex formation between polβ and XRCC1 disease-associated variants similar to the wild-type proteins.

In order to validate the polβ/XRCC1 binding in the presence of the variants, we also performed GST pull-down assays where GST-tag polβ or a GST alone was respectively incubated with his-tagged XRCC1 proteins (wild-type or mutants) after permitting the protein–protein interaction to occur and before being precipitated by GST-binding glutathione beads. The bound material was then captured in three independent experimental GST-pull down tests and analyzed on SDS-PAGE (Fig. S2). Our results revealed the interactions between XRCC1 and polβ in the pulled-down complexes with wild-type proteins and the most dramatic effect was observed with the V86R mutant, where polβ retention was equivalent to polβ alone negative control (Fig. S2, *A*–*C*). However, XRCC1 R109A mutant was pulled down by GST-polβ with relatively weak binding affinity (Fig. S2*A*) and showed slight differences with XRCC1 variants P161L, R194W, Y576S, R280H, and R399Q (Fig. S2, *B* and *C*). No coprecipitation of any complexes was observed with GST alone.

### Protein–protein interaction kinetics of XRCC1 with BER proteins

**Figure 2:**
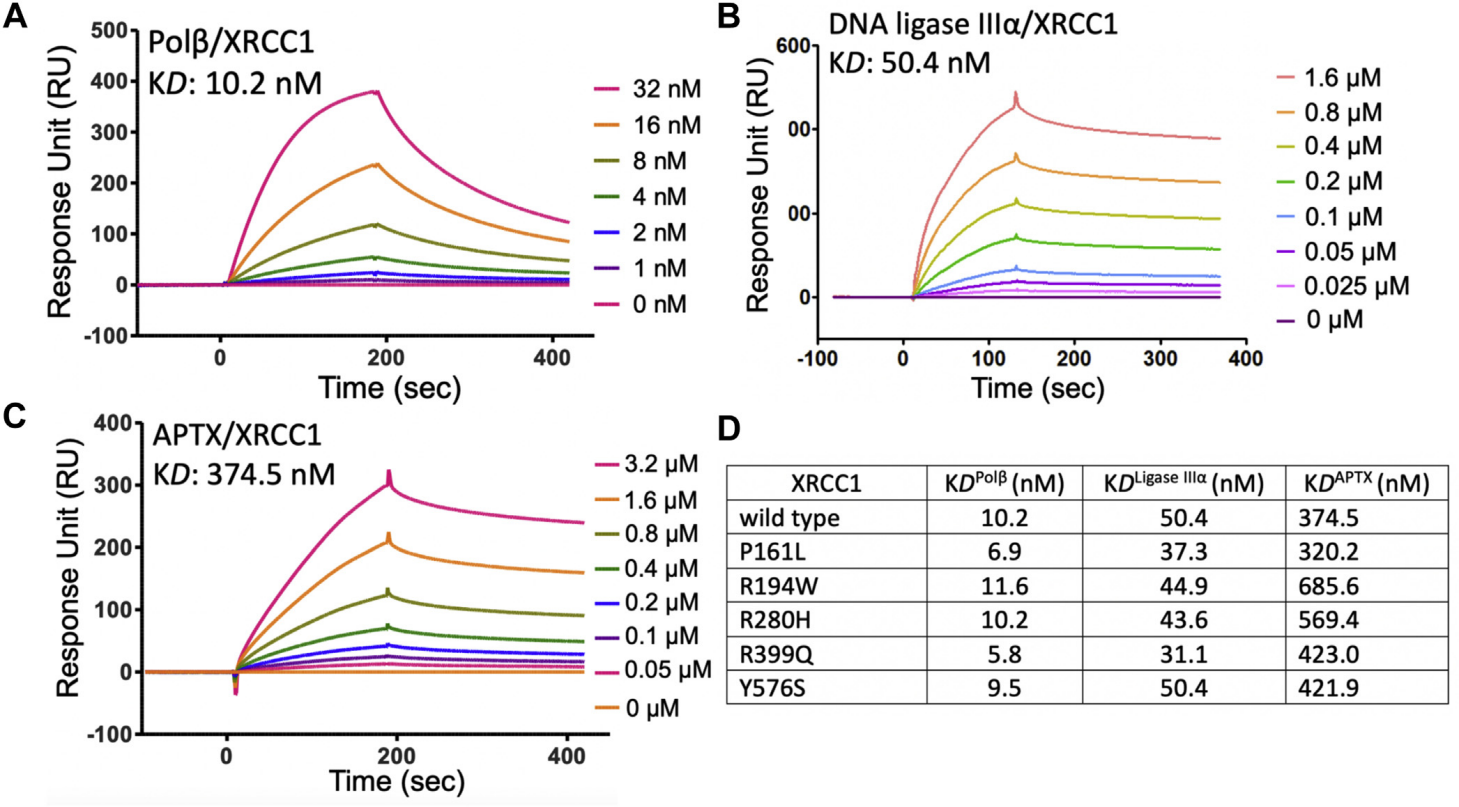
**Protein–protein interaction analyses between XRCC1 and BER proteins.** The real-time protein–protein interaction kinetics of wild-type XRCC1 with polβ (*A*), ligase IIIα (*B*), and APTX (*C*) is measured by SPR assay where the interacting protein partner of XRCC1 was immobilized on CM5 biosensors. The ligand association and dissociation phases are shown for the protein concentration range of XRCC1 on the side of sensorgrams. *D*, table shows comparison of the equilibrium binding constants (K*D*) between XRCC1 wild-type and variants for polβ, ligase IIIα, and APTX. The protein–protein interaction kinetics for XRCC1 variants are presented in Figs. S3–S5.

We quantitatively monitored the real-time kinetics of protein–protein interactions between XRCC1 and the BER proteins (polβ, ligase IIIα, and APTX) by surface plasmon resonance (SPR) assays where the interacting protein partner of XRCC1 was immobilized on CM5 biosensors onto which XRCC1 (wild-type or variant) protein was respectively passed as analytes. Our results with the wild-type XRCC1 (Fig. 2) showed significant differences in the equilibrium binding constants (K*D*) that demonstrate its relative interaction affinity with its repair protein partners polβ (10.2 nM), ligase IIIα (50.4 nM), and APTX (374.5 nM).

For polβ/XRCC1 interaction, when compared with wild-type XRCC1 (Fig. 2*A*, K*D*: 10.2 nM), we observed a significant decrease in polβ-binding affinities for XRCC1/polβ interaction mutants V86R and R109A (K*D*: 284 nM and 170 nM, respectively) as expected (Fig. S3, *A* and *B*). XRCC1 cancer-associated variants P161L, R194W, R280H, and Y576S showed almost same polβ interaction affinity with the wild-type protein as the K*D* values were in the range of 7 to 10 nM (Fig. S3, *C*–*F*). Interestingly, there was a relatively stronger interaction (K*D*: 5.8 nM) between polβ and XRCC1 mutant R399Q (Fig. S3*G*). Similarly, we observed wild-type level of polβ interaction kinetics with XRCC1 cerebellar ataxia-related mutant K431N (Fig. S3*H*). Our overall results revealed that XRCC1 and its disease-associated variants can interact with polβ (Fig. 2*D*).

We also evaluated the protein–protein interaction kinetics of XRCC1 with the BER proteins DNA ligase IIIα and APTX that play roles at the downstream steps of the repair pathway to ligate the final polβ repair product and correct the ligation failure intermediates, respectively. For DNA ligase IIIα/XRCC1 interaction, when compared with wild-type XRCC1 (Fig. 2*B*, K*D*: 50.4 nM), the binding constant values for the cancer-associated variants P161L, R194W, R280H, R399Q, and Y576S showed similar interaction patterns as the K*D* values were in the range of 30 to 50 nM (Fig. S4, *A*–*E*). This was also the case for polβ interaction mutants V86R and R109A as expected (Fig. S4, *F* and *G*). For APTX/XRCC1 interaction, our results showed significantly reduced binding affinity of wild-type XRCC1 for this DNA-end processing enzyme (Fig. 2*C*, K*D*: 374.5 nM) when compared with the other BER proteins tested in this study (Fig. 2*D*). We obtained little or no effect on the equilibrium constant (K*D*: ∼ 300–500 nM) with the cerebellar ataxia-related XRCC1 K431N mutant and the cancer-associated variants P161L, R194W, R280H, R399Q, and Y576S (Fig. S5). Our overall results revealed that XRCC1 can interact with ligase IIIα and APTX with ∼5- and 50-fold lower binding affinities, respectively, when compared with stronger protein–protein interactions we observed between polβ and XRCC1. For all three BER protein partners, there is no significant difference between XRCC1 wild-type and disease-associated variants in the interaction profiles (Fig. 2*D*).

### DNA-binding affinities of XRCC1 to gap and nick repair intermediates

**Figure 3:**
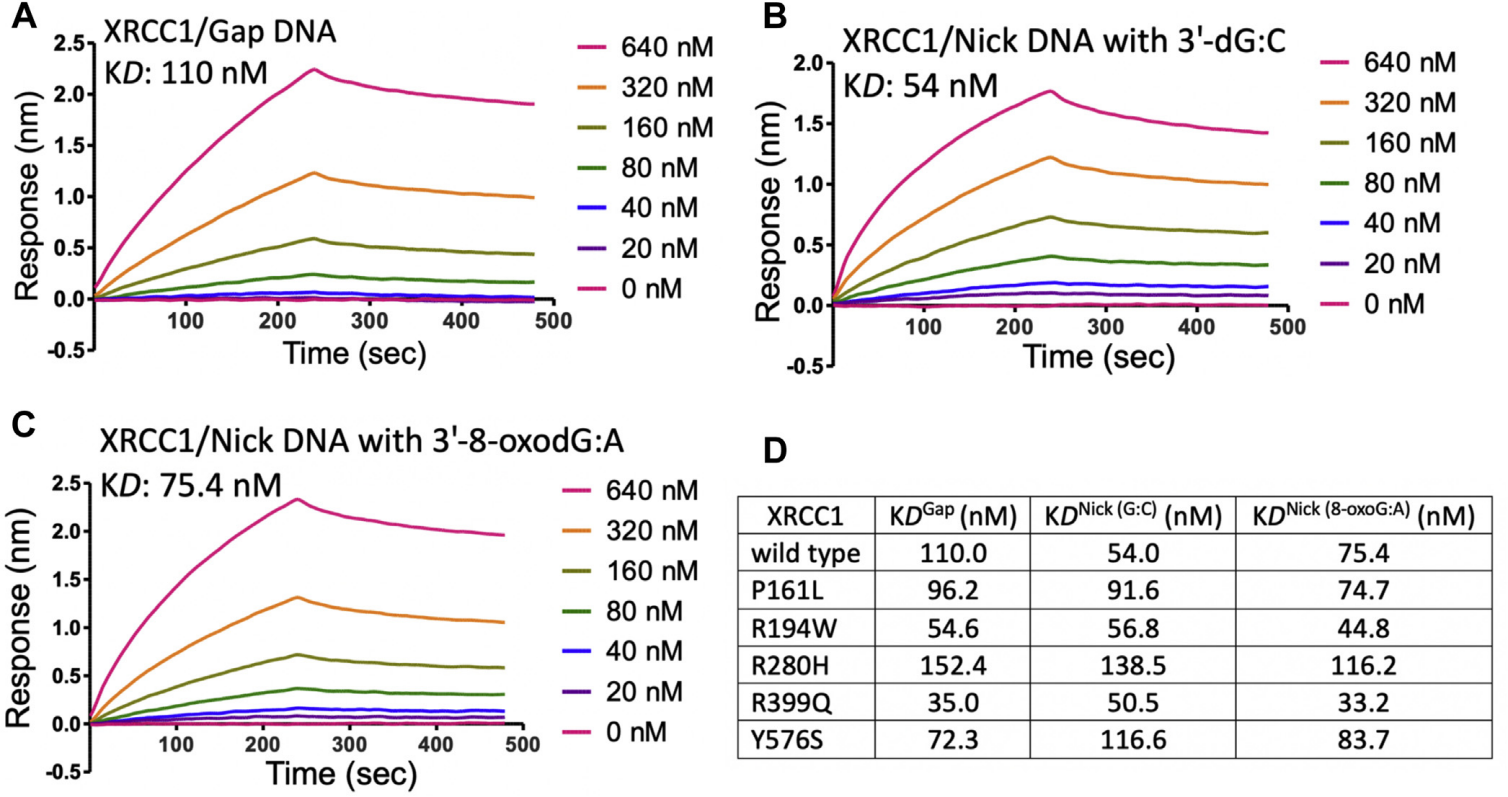
**DNA-binding kinetics of XRCC1.** The real-time DNA-binding kinetics of wild-type XRCC1 to one nucleotide gap (*A*) and nick DNA with 3′-dG:C (*B*) or 3′-8-oxodG:A (*C*). The sensorgrams are shown for the concentrations range of XRCC1 (0–640 nM) where the DNA with a biotin label is immobilized on the streptavidin biosensors. *D*, table shows comparison of the equilibrium binding constants (K*D*) between XRCC1 wild-type and variants for gap and nick DNA with and without damaged ends. The real-time DNA-binding measurements for XRCC1 variants are presented in Figs. S6, S8, and S9.

We then examined DNA-binding affinities of XRCC1 wild-type and cancer-associated variants P161L, R194W, R280H, R399Q, and Y576S in real time using the BLI assay. For this purpose, we used one nucleotide gap DNA and nick DNA with preinserted 3′-dG:C and 3′-8-oxodG:A that mimic the repair intermediates that polβ and ligase IIIα use during gap filling and subsequent nick sealing steps, respectively. Our results demonstrated that the wild-type XRCC1 (Fig. 3) shows relatively stronger binding affinity to both nick DNA substrates (K*D*: 54 or 75.4 nM) than that of gap DNA (K*D*: 110 nM).

For one nucleotide gap DNA-binding affinity of XRCC1 wild-type (Fig. 3*A*) and the cancer-associated variants (Fig. S6), P161L and Y576S, showed similar K*D* values that were in the range of 70 to 100 nM. However, we observed tighter gap DNA binding with XRCC1 variants R194W and R399Q (K*D*: ∼30–50 nM), while R280H (K*D*: ∼153 nM) exhibits slightly lower affinity. In the control experiments, we obtained ∼20-fold difference in the binding affinities between polβ (K*D*: 5.4 nM) and XRCC1 (K*D*: 110 nM) for one nucleotide gap repair intermediate (Fig. S7). For nick DNA-binding affinity of XRCC1 wild-type (Fig. 3*B*) and the cancer-associated variants (Fig. S8) to the repair intermediate with correctly base-paired 3′-dG:C ends, we observed similar K*D* values that were in the range of ∼50 to 60 nM with XRCC1 variants R194W and R399Q, while P161L and Y576S (K*D*: ∼90–110 nM) show a relatively lower affinity constant. However, there was approximately twofold higher K*D* with R280H variant (∼140 nM) in comparison with the wild-type and all other XRCC1 mutants tested in this study (Fig. 3*D*). In the control experiments, we obtained approximately tenfold difference in the equilibrium binding constant between ligase IIIα (K*D*: ∼5 nM) and XRCC1 (K*D*: ∼60 nM) for the nick DNA with 3′-dG:C (Fig. S7). For nick DNA-binding affinity of XRCC1 wild-type (Fig. 3*C*) and the cancer-associated variants (Fig. S9) to the repair intermediate with oxidatively damaged 3′-8-oxodG:A ends, we also obtained a decrease in the nick-binding affinity of XRCC1 variant R280H (K*D*: 116 nM), while all other mutants exhibited wild-type level of K*D* values (Fig. 3*D*). This could be because of significant differences in the association and dissociation rates of the XRCC1 variants for the gap and nick repair intermediates with undamaged *versus* damaged ends tested in this study (Table S4). Our overall results demonstrated that XRCC1 wild-type and all mutants can bind to the one nucleotide gap and nick DNA repair intermediates and their binding affinities are lower than those of polβ and ligase IIIα, respectively (Fig. 3*D*).

### Ligation of polβ nucleotide insertion products by DNA ligase IIIα in the absence and presence of XRCC1

**Figure 4:**
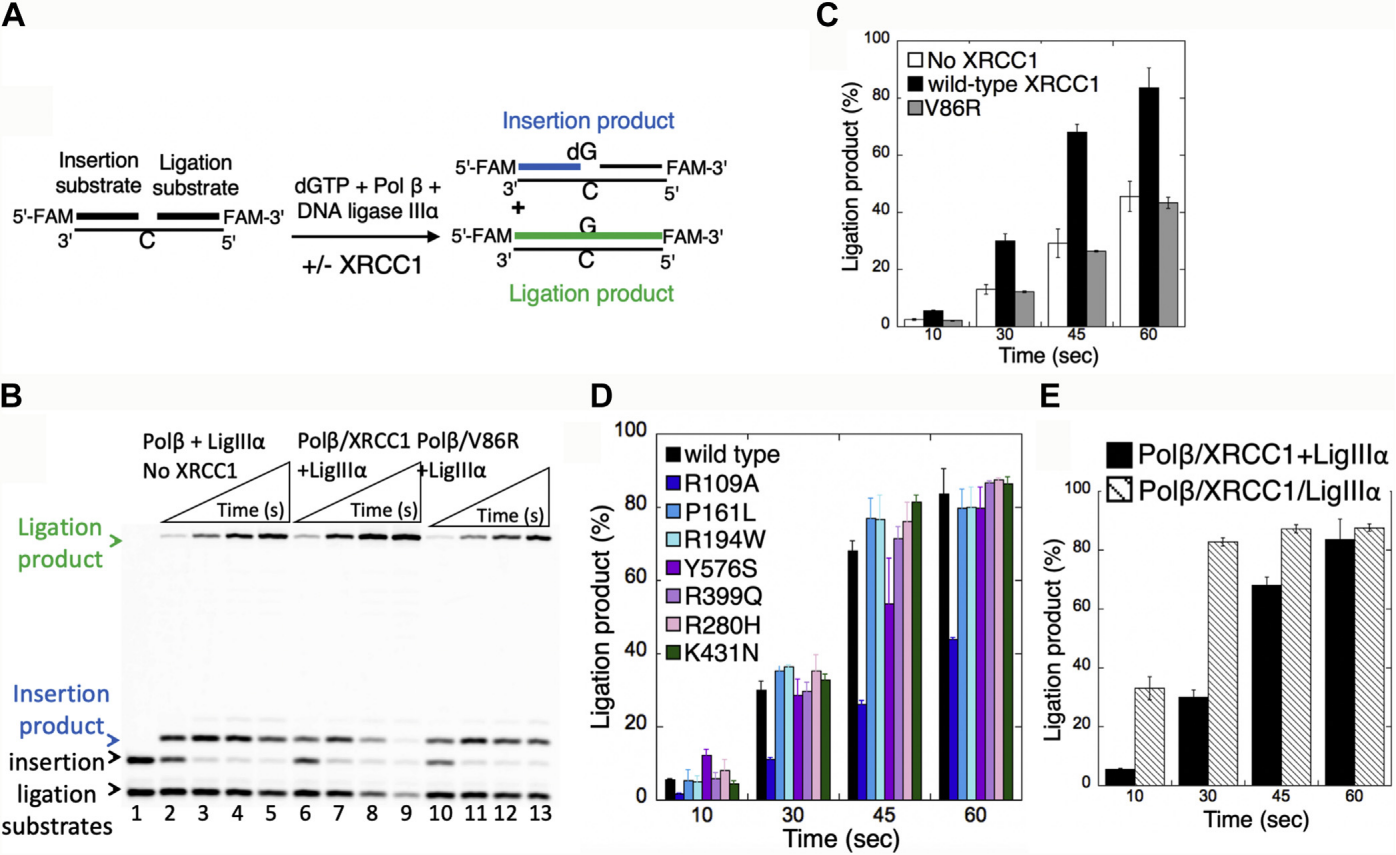
**Ligation of polβ dGTP insertion products by ligase IIIα in the presence of XRCC1.***A*, illustration of the one nucleotide gap DNA substrate with template base C and the insertion and ligation products observed in the coupled assays including polβ, ligase IIIα, and/or XRCC1. *B*, line 1 is the negative enzyme control of the one nucleotide gap DNA substrate with template C. Lanes 2 to 5 are the ligation of polβ dGTP:C insertion products by ligase IIIα in the absence of XRCC1. Lanes 6 to 9 and 10 to 13 are polβ dGTP:C insertion coupled to ligation products in the presence of XRCC1 wild-type and V86R mutant, respectively, and correspond to time points of 10, 30, 45, and 60 s. *C*–*E*, the graph shows time-dependent changes in the amount of ligation products in the absence or presence of XRCC1 (*C*), disease-associated variants (*D*), and the polβ/XRCC1/ligase IIIα complex (*E*). The data represent the average of three independent experiments ±SD. The gel images for XRCC1 variants and the complex are presented in Fig. S10. The bar graphs with individual data points are presented in Fig. S11.

In addition to the protein complex formation, protein–protein interaction, and DNA-binding measurements with XRCC1 wild-type and disease-related variants (Figure 1, Figure 2, Figure 3), in the present study, we also analyzed the effect of XRCC1 on the substrate-product channeling from polβ to ligase IIIα at the downstream steps of the repair pathway in reconstituted BER reactions *in vitro*. For this purpose, we used one nucleotide gap DNA substrate with template C and performed the coupled repair assays that measure polβ nucleotide insertion coupled to DNA ligation at the same time points of incubation in a reaction mixture including the polβ/XRCC1 complex, ligase IIIα, and dGTP. Using this assay, we compared the ligation of polβ dGTP insertion products by ligase IIIα in the absence and presence of XRCC1 (Fig. 4*A*).

For the reaction mixtures containing polβ, dGTP, and ligase IIIα, the results showed the time courses of product formation for single nucleotide gap filling (*i.e.*, polβ dGTP:C insertion products) and final DNA ligation (*i.e.*, nick sealing of polβ dGMP:C insertion products). Without XRCC1, we observed an increase in both gap filling and ligation products as a function of incubation time (Fig. 4*B*, lanes 2–5). With addition of XRCC1, more ligation product was observed along with simultaneous disappearance with the gap filling product of BER intermediate (Fig. 4*B*, lanes 6–9), suggesting that XRCC1 facilitates the conversion of polβ dGTP:C insertion products to the complete ligated products. The amount of ligation products showed approximately twofold increase (Fig. 4*C*). In the control experiments including polβ interaction-deficient mutant of XRCC1 V86R (Fig. 4*B*, lanes 10–13), we obtained similar results with the amount of ligation products in the absence of XRCC1 (Fig. 4*C*).

We also tested the impact of XRCC1 variants on the ligation efficiency after polβ dGTP:C insertions. With the addition of XRCC1 R109A mutant that is known to have a destabilized polβ interaction, the polβ products of dGTP:C insertion were not efficiently converted to complete ligation products by ligase IIIα (Fig. S10*A*, lanes 2–5). There was relatively lower amount of ligation products in the presence of R109A mutant when compared with those with wild-type XRCC1 (Fig. 4*D*). However, we obtained an increase in the amount of ligation products over the time of reaction incubation in the presence of the cancer-associated (P161L, R194W, R280H, R399Q, Y576S) and cerebellar ataxia-related K431N XRCC1 variants (Figs. S10, *A*–*C* and S11). These ligation products exhibit no significant difference between the XRCC1 mutants, which were also similar with that of wild-type protein (Fig. 4*D*). Moreover, we obtained enhanced ligation products at the initial time points of coupled reaction when we start the reaction by the addition of preincubated enzyme mixture including all three repair proteins polβ/XRCC1/ligase IIIα (Fig. S10*C*, lanes 10–13 and Fig. S11) in comparison with the reaction that was started with the addition of polβ/XRCC1 complex (Fig. 4*E*). In agreement with previously reported data (35, 36, 37, 38), our overall results indicate that XRCC1 stimulates the processivity of a BER reaction after polβ gap filling and the channeling of the nick repair product (polβ dGTP:C insertion) to the final step for its ligation by ligase IIIα in the coordinated repair pathway.

**Figure 5:**
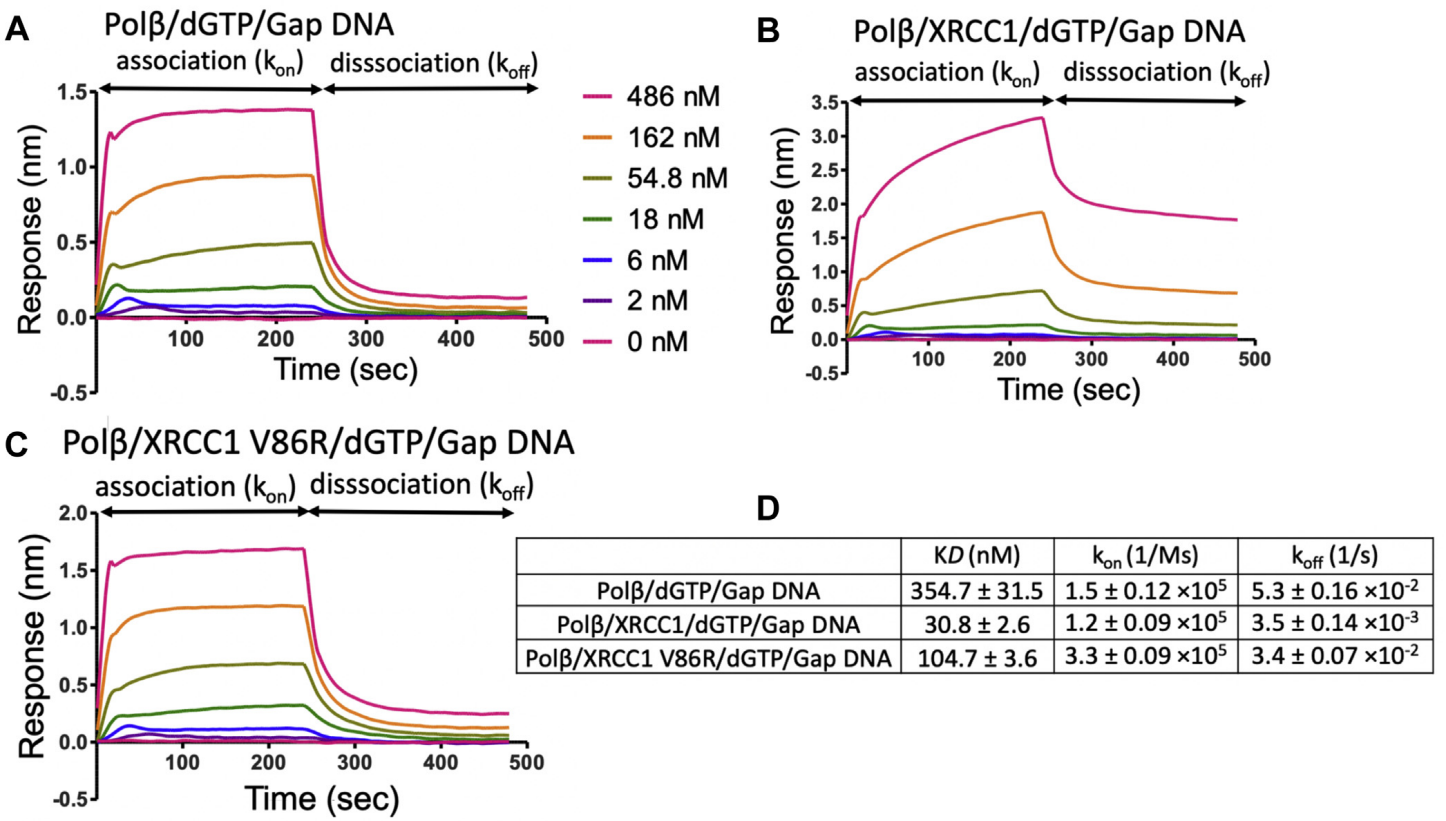
**Gap DNA-binding kinetics of polβ/XRCC1 complex in the presence of dGTP.** The real-time DNA-binding kinetics of polβ/dGTP to one nucleotide gap DNA with template base C in the absence (*A*) and presence (*B*) wild-type XRCC1 and polβ/XRCC1 interaction mutant V86R (*C*). *D*, table shows the effect of XRCC1 on the equilibrium binding constant (K*D*), the association (k_on_) and dissociation (k_off_) rates of polβ/dGTP/gap DNA catalytic ternary complex. The data are processed and analyzed with ForteBio data analysis software with 1:1 binding model.

In order to further understand this XRCC1-mediated handoff process, we monitored the real-time kinetics of binding *versus* dissociation rates of polβ from one nucleotide gap DNA using the catalytic enzyme in the presence of dGTP (polβ/dGTP/gap DNA) and compared the kinetic parameters of this ternary complex in the absence and presence of XRCC1 (Fig. 5). Our results demonstrated that polβ dissociates faster (k_off_: 5.3 × 10^−2^) in the absence of XRCC1 (Fig. 5*A*). The addition of XRCC1 enhances the stability of the catalytic ternary complex, and the dissociation rate of polβ (k_off_: 3.5 × 10^−3^) from gap DNA in the presence of XRCC1 was slower (Fig. 5*B*). We observed ∼12-fold difference in the equilibrium constant (K*D*: ∼355 *versus* ∼30 nM, Fig. 5*D*). Yet, this stabilizing effect of XRCC1 on tight binding of polβ to gap DNA was deficient in the presence of XRCC1 mutant V86R with diminished polβ interaction site (Fig. 5*C*). Our findings suggest that polβ should be preferentially positioned and locked at the 3′-end of the primer strand and XRCC1 could stabilize polβ on the nick repair intermediate with an inserted dGMP (*i.e.*, 3′-dG:C) to which DNA ligase can bind and ligate during next nick sealing step in the coordinated repair pathway.

### Ligation of polβ oxidized nucleotide insertion products by DNA ligase IIIα in the absence and presence of XRCC1

**Figure 6:**
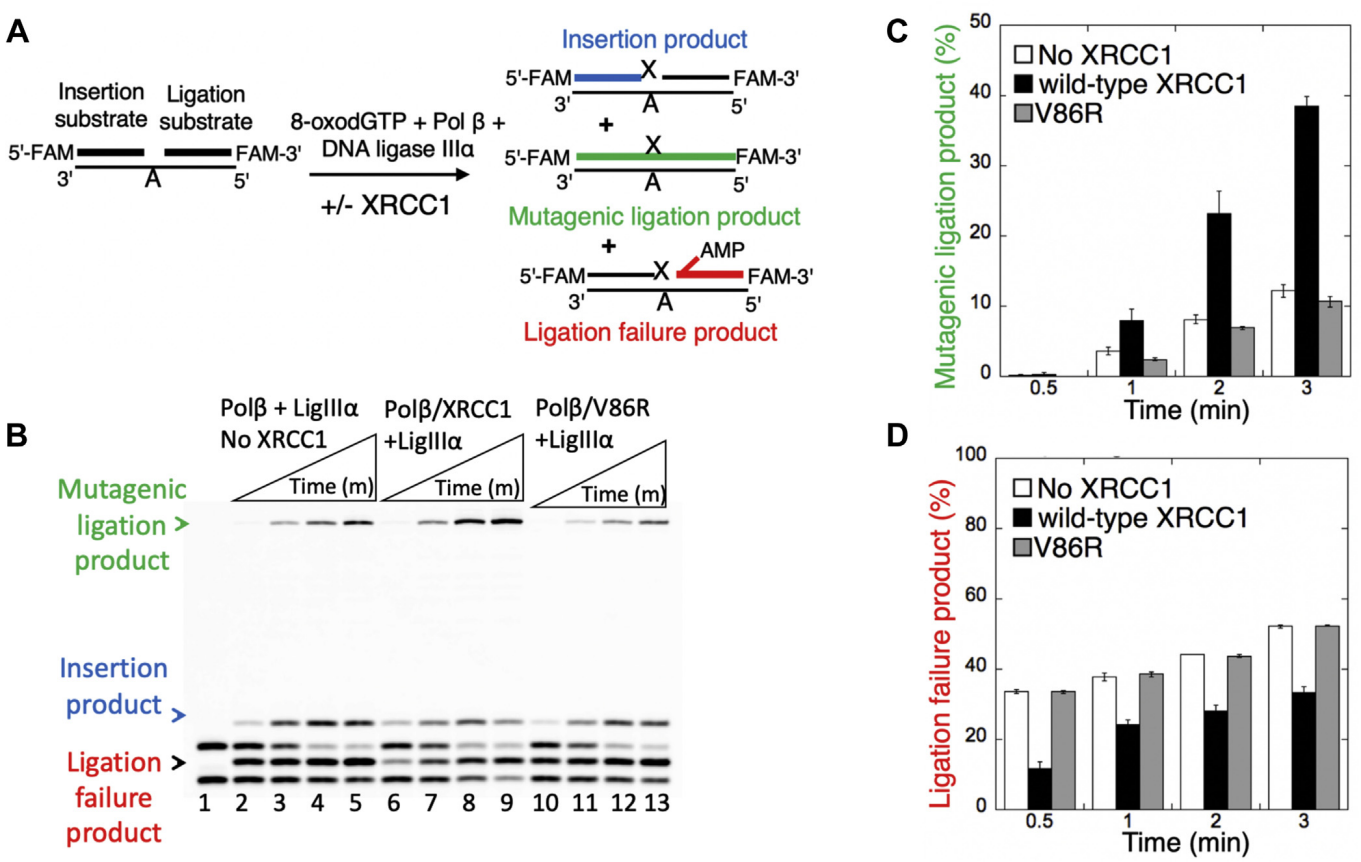
**Ligation of polβ 8-oxodGTP insertion products by ligase IIIα in the presence of XRCC1.***A*, illustration of the one nucleotide gap DNA substrate and the insertion, mutagenic ligation, and ligation failure products observed in the coupled assays including polβ, ligase IIIα, and/or XRCC1. *B*, line 1 is the negative enzyme control of the one nucleotide gap DNA substrate with template A. Lanes 2 to 5 are the ligation of polβ 8-oxodGTP:A insertion products by ligase IIIα in the absence of XRCC1. Lanes 6 to 9 and 10 to 13 are polβ 8-oxodGTP:A insertion coupled to ligation products in the presence of XRCC1 wild-type and V86R mutant, respectively, and correspond to time points of 0.5, 1, 2, and 3 min. *C* and *D*, the graphs show time-dependent changes in the amount of mutagenic ligation (*C*) and ligation failure (*D*) products. The data represent the average of three independent experiments ±SD. The bar graphs with individual data points are presented in Fig. S12.

In our previous studies, we reported that DNA ligation step of the BER pathway is compromised after polβ 8-oxodGTP insertion leading to the formation of ligation failure products with 5′-adenylate (AMP), which could lead to the formation of toxic strand break intermediates and more cytotoxicity in polβ^+/+^ cells than polβ^−/−^ cells (67). In the present study, we also analyzed the effect of XRCC1 on the substrate-product channeling after polβ oxidized nucleotide insertion in a reconstituted BER reaction including one nucleotide gap DNA substrate with template A, polβ/XRCC1 complex, ligase IIIα, and 8-oxodGTP (Fig. 6*A*). In the absence of XRCC1 (Fig. 6*B*, lanes 2–5), the results showed the products formation for single nucleotide gap filling (*i.e.*, polβ 8-oxodGTP insertion products), mutagenic ligation (*i.e.*, nick sealing of an inserted 8-oxodGMP insertion products), and ligation failure with 5′-AMP. With the addition of XRCC1 (Fig. 6*B*, lanes 6–9), more mutagenic ligation product was observed (approximately fourfold increase) along with simultaneous conversion of polβ 8-oxodGTP insertion products, which was accompanied with a decrease in the ligation failure products (Fig. 6, *C* and *D* and Fig. S12). In the control experiments including polβ interaction-deficient mutant of XRCC1 V86R, we obtained the similar results with the reaction in the absence of XRCC1 (Fig. 6*B*, lanes 10–13).

**Figure 7:**
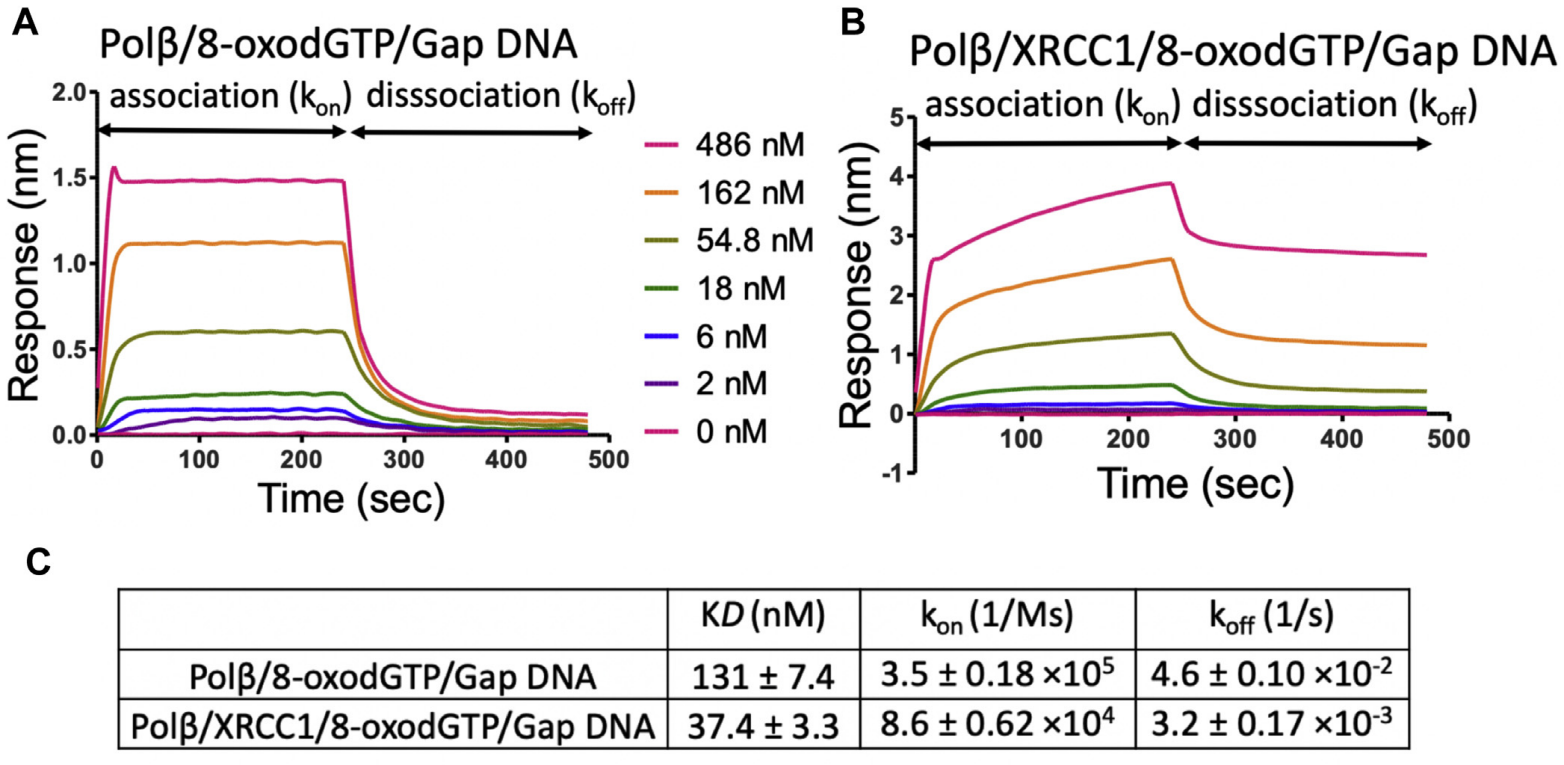
**Gap DNA-binding kinetics of polβ/XRCC1 complex in the presence of 8-oxodGTP.** The real-time binding kinetics of polβ/8-oxodGTP to one nucleotide gap DNA with template base A in the absence (*A*) and presence (*B*) of XRCC1. *C*, table shows the effect of XRCC1 on equilibrium binding constant (K*D*), the association (k_on_) and dissociation (k_off_) rates of polβ/8-oxodGTP/gap DNA catalytic ternary complex. The data are processed and analyzed with ForteBio data analysis software with 1:1 binding model.

We also performed the real-time kinetics assays to measure the binding *versus* dissociation rates of polβ in the presence of 8-oxodGTP using the catalytic enzyme complex (polβ/8-oxodGTP/gap DNA with template base A) and compared the kinetic parameters of this ternary complex in the absence and presence of XRCC1 (Fig. 7). Similar to the ternary complex including correct nucleotide (polβ/dGTP/gap DNA with template base C), we obtained a slower dissociation rate of polβ in the presence of 8-oxodGTP from gap DNA (k_off_: 3.2 × 10^−3^) by the effect of XRCC1 (Fig. 7, *A* and *B*). In this case, we observed approximately fourfold difference in the equilibrium binding constant (Fig. 7*C*).

### Ligation of nick DNA by ligase IIIα in the absence and presence of XRCC1

**Figure 8:**
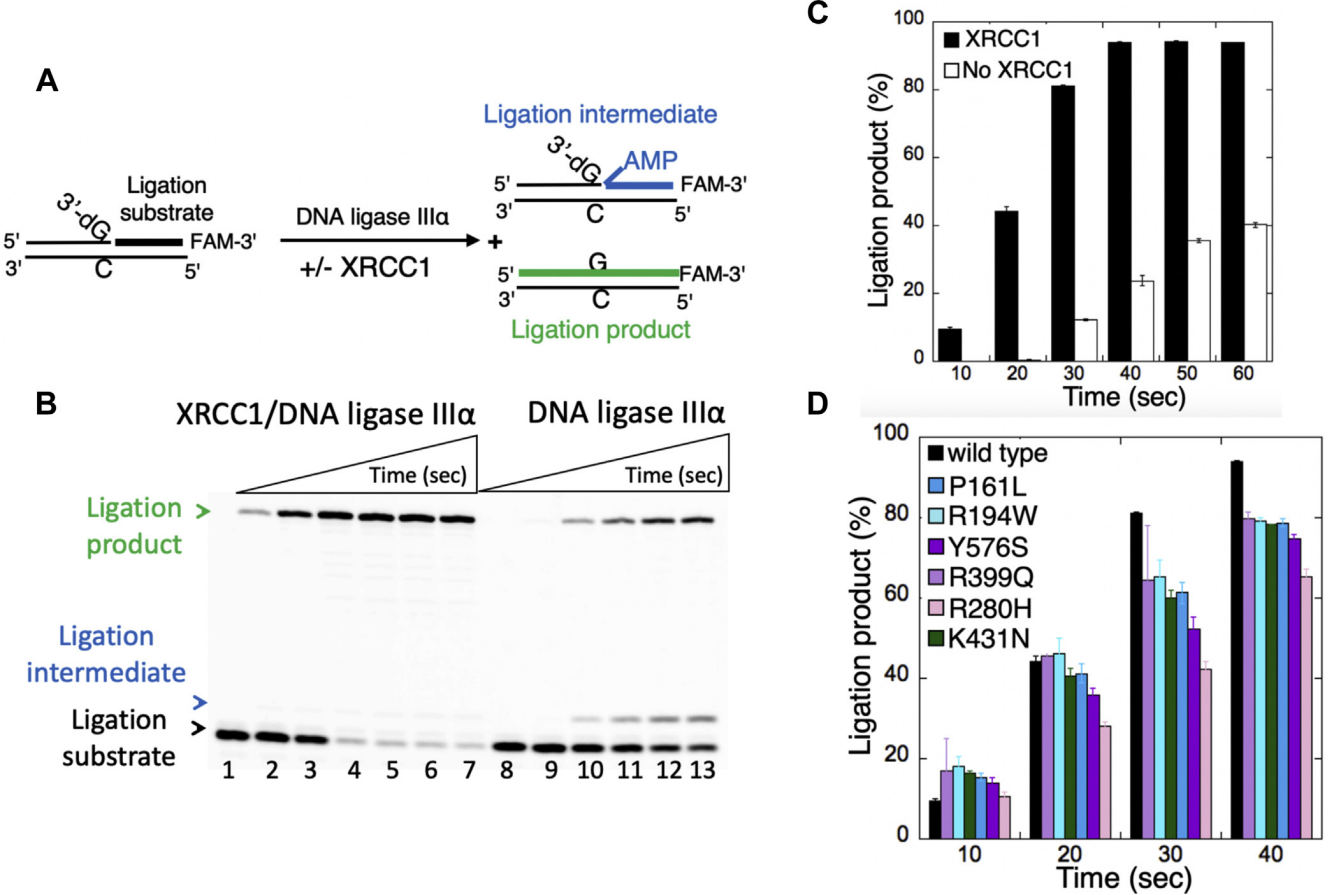
**Ligation of nick repair intermediate by ligase IIIα in the presence of XRCC1.***A*, illustrations of the nick DNA substrate with preinserted 3′-dG:C, the ligation reaction intermediates with 5′-adenylate (AMP), and ligation products. *B*, line 1 is the negative enzyme control of the nick DNA substrate. Lanes 2 to 7 and 8 to 13 are the ligation products in the presence and absence of XRCC1, respectively, and correspond to time points of 10, 20, 30, 40, 50, and 60 s. *C* and *D*, the graphs show time-dependent changes in the amount of ligation products in the absence and presence of XRCC1 (*C*) and disease-associated variants (*D*). The data represent the average of three independent experiments ±SD. The gel images for XRCC1 variants are presented in Fig. S13. The bar graphs with individual data points are presented in Fig. S14.

In addition to the BER assays that mimic the channeling of repair intermediates from polβ to ligase IIIα at the downstream steps, in the present study, we also analyzed the effect of XRCC1 on the nick sealing activity of ligase IIIα at the last ligation step of BER pathway *in vitro*. For this purpose, we used the nick DNA substrate with preinserted 3′-dG:C that mimics polβ dGTP:C insertion product (Fig. 8*A*).

For the ligation reactions containing ligase IIIα alone, the results showed the product formation for nick sealing along with the ligation reaction intermediates with 5′-AMP (Fig. 8*B*, lanes 9–13). The addition of XRCC1 stimulates the end joining of the nick repair intermediate by ligase IIIα (Fig. 8*B*, lanes 2–7). We observed approximately fourfold increase in the amount of ligation products with a significant decrease in the reaction intermediates (Fig. 8*C* and Fig. S14). These results suggest that XRCC1 could serve as a facilitatory factor moving the ligation reaction forward.

We also evaluated the XRCC1 cancer-associated (P161L, R194W, R280H, R399Q, and Y576S) and the cerebellar ataxia-related (K431N) variants to compare their impact on the ligation efficiency of nick repair intermediate by ligase IIIα. Overall results demonstrated the formation of ligation products over the time of reaction incubation (Figs. S13 and S14) and slight differences between XRCC1 mutant proteins (Fig. 8*D*). Interestingly, we observed relatively diminished end-joining activity of ligase IIIα in the ligation reaction including XRCC1 R280H variant that exhibits lowest nick DNA-binding affinity over all other XRCC1 mutants tested in this study (Fig. 3*D*).

**Figure 9:**
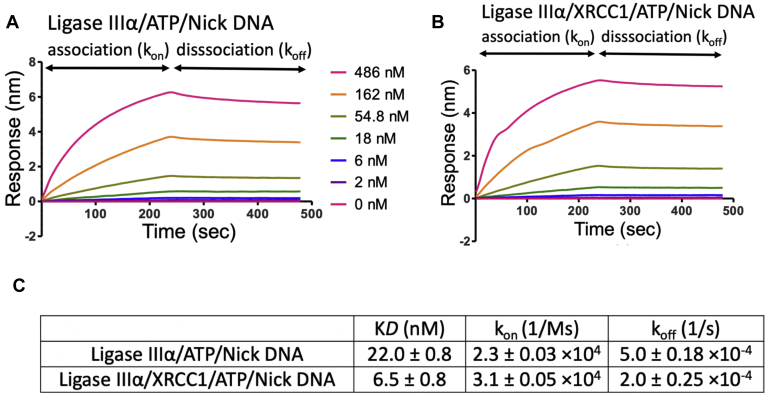
**Nick DNA-binding kinetics of ligase IIIα/XRCC1 complex in the presence of ATP.** The real-time binding kinetics of ligase IIIα/ATP to nick DNA in the absence (*A*) and presence (*B*) of XRCC1. *C*, table shows the effect of XRCC1 on the equilibrium binding constant (K*D*), the association (k_on_) and dissociation (k_off_) rates of ligase IIIα/ATP/nick DNA catalytic ternary complex.

In order to further understand this XRCC1-enhanced ligation of nick DNA with preinserted 3′-dG:C, we monitored the real-time kinetics of nick DNA binding and measured association *versus* dissociation rates of ligase IIIα in the catalytic complex including ATP (ligase IIIα/ATP/nick DNA) in the absence *versus* presence of XRCC1 (Fig. 9). Our results demonstrated that ligase IIIα dissociates from nick DNA faster (k_off_: 5.0 × 10^−4^) in the absence of XRCC1 (Fig. 9*A*) and XRCC1 stabilizes the complex (Fig. 9*B*) at slower ligase dissociation rate (k_off_: 2.0 × 10^−4^). We observed approximately fourfold difference in the equilibrium binding constant (K*D*: 6.5 *versus* 22 nM) with a tighter binding affinity in the presence of XRCC1 (Fig. 9*C*).

### Impact of XRCC1/ligase IIIα interaction domain on the efficiency of downstream steps in BER pathway

**Figure 10:**
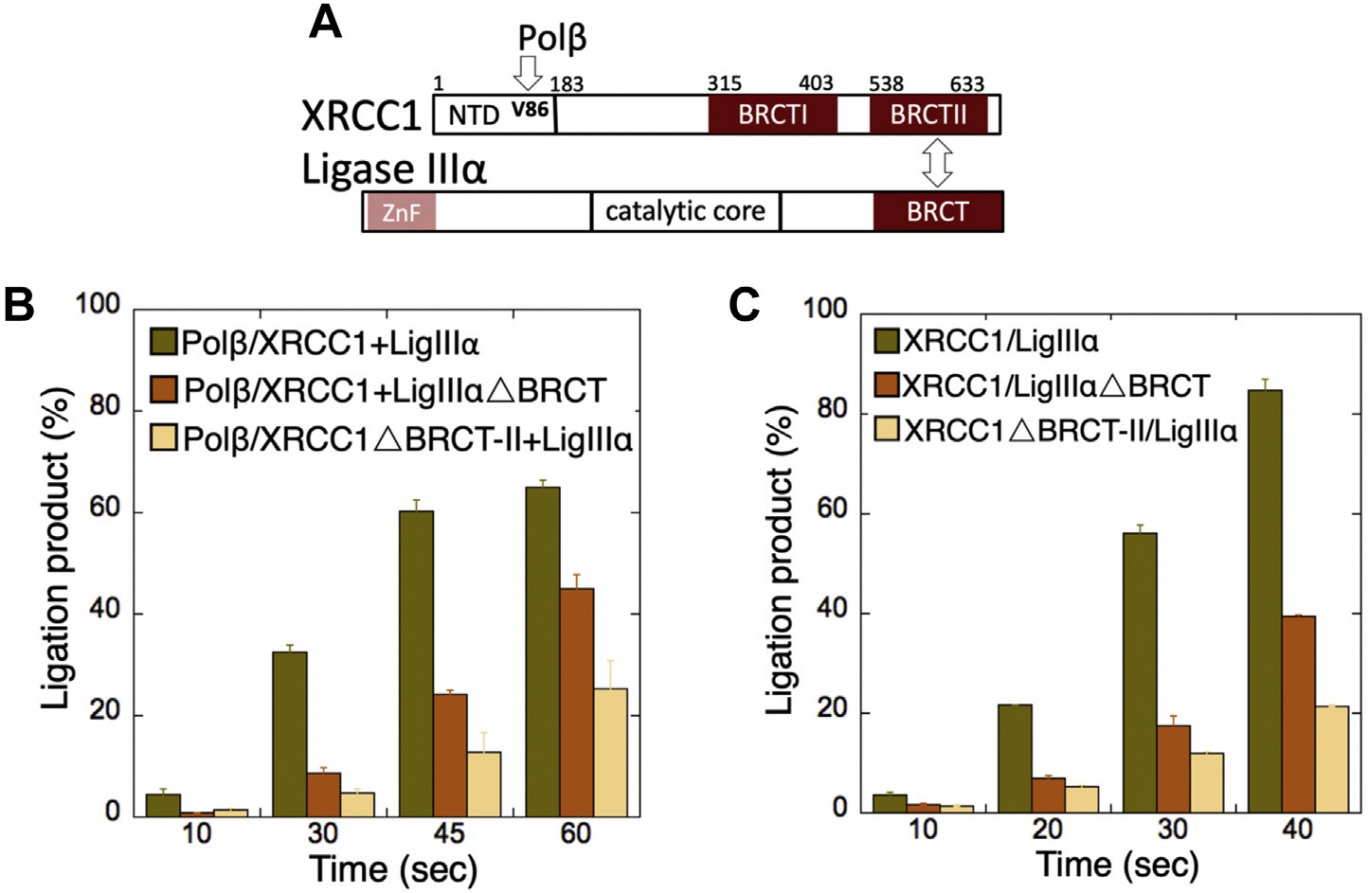
**Impact of XRCC1/ligase IIIα interacting BRCT domains on the ligation of polβ nucleotide insertion products and nick sealing.***A*, the interaction regions of XRCC1 with polβ and ligase IIIα. *B* and *C*, the graphs show time-dependent changes in the amount of ligation products in the presence of XRCC1/ligase IIIα, XRCC1/ligase IIIαΔBRCT, and XRCC1ΔBRCT-II/ligase IIIα. The data represent the average of three independent experiments ±SD. The gel images are presented in Fig. S15. The bar graphs with individual data points are presented in Fig. S16.

We then evaluated the effect of BRCT domains that mediate the protein–protein interaction between XRCC1 and DNA ligase IIIα using the truncated proteins ligase IIIα▵BRCT and XRCC1▵BRCT-II (Fig. 10*A*). For this purpose, we first tested the effect of BRCT domains on the channeling of polβ dGTP:C insertion products in the coupled assays as described above (Fig. 4*A*). We observed a decrease in the conversion of polβ dGTP:C insertion products to complete ligated products in the reaction including polβ, full-length XRCC1, and ligase IIIα▵BRCT (Fig. S15*A*, lanes 6–9). This decrease was more significant when we tested the ligation of polβ dGTP:C insertion products in the coupled reaction containing XRCC1▵BRCT-II and full-length ligase IIIα (Fig. S15*A*, lanes 10–13). In both cases, polβ gap filling products were accumulated in the reaction due to inefficient handoff of these repair intermediates to final ligation step. Overall, when compared with the coupled reaction including full-length proteins (Fig. S15*A*, lanes 2–5), there was ∼4- to 8-fold decrease in the amount of ligation products as a function of incubation time for the BRCT-domain deficient proteins of XRCC1 and ligase IIIα (Fig. 10*B* and Fig. S16).

We finally compared the effect of BRCT domains on the nick sealing ability of ligase IIIα in the ligation reactions as described above (Fig. 8*A*). The ligation of the nick DNA with 3′- dG:C by ligase IIIα in the presence of ligase IIIα without XRCC1 interaction region (ligase IIIα▵BRCT) showed lower amount of nick sealing products (Fig. S15*B*, lanes 6–9) when compared with the full-length proteins (Fig. S15*B*, lanes 2–5). Moreover, the end-joining ability of full-length ligase IIIα was diminished in the presence of XRCC1 without ligase IIIα interaction region (XRCC1▵BRCT-II) (Fig. S15*B*, lanes 10–13). The amount of ligation products was significantly lower when compared with the ligation products of full-length proteins (Fig. 10*C* and Fig. S16).

## Discussion

BER is required for the repair of majority of endogenous DNA damages (1, 2, 3, 4, 5). BER deficiency in mouse models and the defects in crucial repair proteins involving in short-patch BER have been associated with neurological disorders and cancer as shown in the functional studies with BER polymorphisms (68). For example, germline and tumor-associated variants that have been identified in polβ, the main and error-prone polymerase involved in BER, have functional phenotypes associated with lung, gastric, colorectal, and prostate cancer (69). Similarly, a large number of germline and cancer-associated SNP variants that affect amino acid composition of XRCC1 have been reported in the human population, and epidemiology studies have found an association of some of these with cancer risk (59, 60, 61, 62, 63, 64, 65, 66, 70, 71, 72, 73, 74). Furthermore, the presence of XRCC1 germline polymorphisms has also been found to be associated with responses to environmental exposures of toxicants (75, 76, 77, 78, 79, 80, 81). For example, the individuals exposed to chromium and the smokers who carry XRCC1 Arg399Gln (R399Q) variant exhibit significantly increased numbers of aberrations in their lymphocytes, deficiency in the ability to repair ionizing radiation damage, and increased frequencies of micronuclei and chromosomal aberrations (70, 71, 72, 73, 74, 75, 76, 77, 78, 79, 80, 81).

BER involves a substrate-product channeling mechanism that entails a coordinated handoff from a single nucleotide insertion by polβ into gap to the sealing of 5′- and 3′-DNA ends of the resulting nick repair product by ligase IIIα during gap filling and ligation steps, respectively, in the coordinated repair pathway (21, 22). The important role for XRCC1/polβ interaction for coordinating the efficiency of the BER process in the repair of AP-sites and the stimulatory role of XRCC1 in the ligation reaction by ligase IIIα have been previously reported (20, 33, 34, 35, 36, 37, 38, 40). Yet, the mechanism by which XRCC1, as nonenzymatic scaffold protein, orchestrates the substrate-product channeling from polβ to ligase IIIα particularly at the downstream steps through its protein interactions with both repair enzymes remains undefined. Similarly, the impact of disease-associated mutations affecting the scaffolding function of XRCC1 on this handoff process for the ligation of polβ nucleotide insertion products and nick sealing ability of ligase IIIα has never studied before.

In agreement with previously reported data (33, 34, 35, 36, 37, 38), our results indicate a role of XRCC1 scaffolding function on more stable protein complex formation with polβ, which could lead to an efficient substrate-product channeling to next ligation step and promote concerted repair events to prevent accumulation of cytotoxic repair intermediates. According to the NMR and structural studies, NTD of XRCC1 interacts with polβ/gap DNA binary complex while making contacts with both the C (catalytic/palm) and N (nucleotide binding/thumb) subdomains of the polymerase, and NTD will not interfere with the conformational changes that polβ undergoes for DNA synthesis (82, 83). Our findings revealed that XRCC1 can enhance the gap DNA-binding affinity of polβ within the catalytic repair protein/DNA complex with an incoming correct or oxidized nucleotide. We suggest that XRCC1 does not directly affect polβ gap filling activity for a nucleotide insertion, but instead it stabilizes the enzyme on the repair intermediate during which polβ undergoes a conformational change upon binding of an incoming nucleotide, and this could promote its efficient handoff to ligase IIIα for ligation at the downstream steps of BER pathway. This could enable XRCC1 to accelerate a crucial repair step, which is especially important in a case when the polβ DNA synthesis activity is limiting in the cell as reported for many cancer-associated polβ variants (69).

The interaction between polβ and XRCC1 is crucial for polβ recruitment to DNA damage sites (56, 57, 58). Our results revealed a requirement of polβ/XRCC1 interaction for the stable repair protein complex formation, and this is not significantly affected by the disease-associated mutations in XRCC1. The studies based on the sedimentation equilibrium measurements reported that XRCC1 exists as a monomer at lower protein concentrations but forms a dimer at higher protein concentrations (27, 32). According to our SEC results, wild-type XRCC1 and all mutants were higher oligomer with the elution peaks at ∼8.9 ml and heterotetrameric when it forms a complex with polβ as the position of repair protein complex peak was obtained at ∼11.2 ml. We also demonstrated the similar binding affinities of XRCC1 wild-type and disease-associated variants to one nucleotide gap and nick BER intermediates. Furthermore, the studies with wild-type protein have shown that XRCC1 binds to nick and one nucleotide gap DNA tightly in a stoichiometric manner (1:1) with higher affinity than that of the intact duplex with no break and single-stranded oligonucleotide (23, 24, 25, 82, 83). Interestingly, in consistent with our findings that show a significant difference with R280H in comparison with all other XRCC1 variants tested in this study (Fig. 4*D*), it has been reported that polymorphic variant R280H exhibits a decreased retention time from the site of DNA damage induced single-stranded breaks (84, 85). Furthermore, the amino acid substitutions within the central DNA binding domain (219–415 aa) encompassing the first BRCT domain of XRCC1 have been found to disrupt DNA binding *in vitro* and the recruitment of XRCC1 to near-UV micro-irradiated sites of the nuclei is strongly influenced by the region encompassing amino acids 166 to 310 without affecting its initial recruitment, suggesting that the DNA-binding activity of XRCC1 is crucial for efficient DNA damage repair (86).

In addition, our study demonstrates the role of BRCT domains that mediate protein–protein interactions between XRCC1 and ligase IIIα on the substrate-product channeling and nick sealing at the downstream steps of BER pathway. Moreover, the role of PARP-like zinc finger (ZnF) and DNA-binding domain in nick sensing and joining by the catalytic domain of ligase IIIα has been reported in previous studies (87, 88). Lastly, we showed little or no effect on protein–protein interactions between the wild-type and the disease-associated variants (P161L, R194W, R280H, R399Q, K431N, and Y576S) of XRCC1 with key BER proteins ligase IIIα and APTX. Since these amino acid substitutions are expanded and located in different domains of the XRCC1 protein (Scheme S1), they might represent separation-of-function mutants that are deficient in only a single or a number of interactions with many other repair proteins involved in single-strand break or BER as suggested in earlier studies (59, 64). For example, P161L and Y576S variants were found to have wild-type level of protein interactions with the other repair factor PARP1 and DNA replication protein PCNA. In contrast, R194W and R280H variants exhibit no binding with PNKP, and XRCC1 R194W has been reported to be defective in the interaction with DNA glycosylase OGG1 (59, 60, 61, 62, 63, 64, 89, 90). Structure/function studies are required to understand how XRCC1 orchestrates the BER proteins (APE1, polβ, ligase IIIα, APTX) that function all together within a multiprotein/DNA repair complex to facilitate the faithful channeling of DNA repair intermediates. Gaining an understanding of how DNA damage is coordinately repaired can be exploited as novel targets for future rational chemotherapeutic drug design toward enhancing human health (91).

## Experimental procedures

### Protein purifications

Human wild-type full-length (1–335 aa) and C-terminal domain (92–335 aa) of DNA polymerase (pol) β with or without GST-tag (pGEX-6p-1) were overexpressed and purified as previously described (67, 92, 93, 94, 95). Briefly, the recombinant proteins were overexpressed in BL21(DE3)pLysS *E. coli* cells (Invitrogen) in Lysogeny Broth (LB) media at 37 °C for 8 h and induced with 0.5 mM isopropyl β-D-thiogalactoside (IPTG). The cells were then grown overnight at 16 °C. After cell lysis at 4 °C by sonication in the lysis buffer containing 25 mM HEPES (pH 7.5), 500 mM NaCl, 0.1% NP40, and cOmplete Protease Inhibitor Cocktail (Roche), the lysate was pelleted at 16,000 rpm for 1 h and then clarified by centrifugation and filtration. The supernatant was loaded onto a GSTrap HP column (GE Health Sciences) and purified with the elution buffer containing 50 mM Tris-HCl (pH 8.0) and 10 mM reduced glutathione. In order to cleave a GST-tag, the recombinant protein was incubated with PreScission Protease (GE Health Sciences) for 16 h at 4 °C in the buffer containing 1X PBS (pH 7.3), 200 mM NaCl, and 1 mM DTT. After the cleavage, the polβ protein was subsequently passed through a GSTrap HP column, and the protein without GST-tag was then further purified by loading onto Superdex 200 gel-filtration column (GE Health Sciences) in the buffer containing 50 mM Tris-HCl (pH 7.5) and 400 mM NaCl.

Human wild-type full-length DNA ligase IIIα (1–922 aa) was obtained from GenScript and cloned into the pET-29a expression vector (Novagen). Human wild-type truncated protein of DNA ligase IIIαΔBRCT (1–755 aa) was cloned into pET-24b expression vector (Novagen). The recombinant his-tag proteins were overexpressed in BL21(DE3) *E. coli* cells (Invitrogen) in LB media at 37 °C for 8 h and induced with 0.5 mM IPTG. The cells were harvested, lysed at 4 °C, and then clarified as described above. The supernatant was loaded onto a HisTrap HP column (GE Health Sciences) and purified with an increasing imidazole gradient (0–300 mM) elution at 4 °C. The collected fractions were then further purified by Superdex 200 Increase 10/300 chromatography (GE Healthcare) in the buffer containing 50 mM Tris-HCl (pH 7.0), 500 mM NaCl, glycerol 5%, and 1 mM DTT.

Human wild-type full-length XRCC1 (1–633 aa), the truncated proteins XRCC1 N-terminal domain (1–184 aa), and XRCC1ΔBRCT-II (1–535 aa) were cloned into pET-24b expression vector (Novagen). XRCC1 mutants were cloned into pET-24b expression vector (Novagen) using the primers listed in Table S1. The mutations were made to XRCC1 at sites that are important for polβ interaction (V86R and R109A) and the site-specific amino acid variants (P161L, R194W, R280H, R399Q, K431N, Y576S) were created using the Quick-Change II Side-Directed Mutagenesis kit (Stratagene). All plasmids for the XRCC1 mutant constructs were confirmed by DNA sequencing prior to use. His-tagged recombinant XRCC1 proteins were overexpressed in BL21(DE3) *E. coli* cells (Invitrogen), and the cells were harvested, lysed at 4 °C, and then clarified as described above. The supernatant was loaded onto a HisTrap HP column (GE Health Sciences) and purified with an increasing imidazole gradient (0–300 mM) elution at 4 °C. The collected fractions were then subsequently loaded onto a HiTrap Heparin column (GE Health Sciences) with a linear gradient of NaCl up to 1 M. The recombinant XRCC1 proteins were then further purified by Superdex 200 Increase 10/300 chromatography (GE Healthcare) in the buffer containing 50 mM Tris-HCl (pH 7.0), 500 mM NaCl, glycerol 5%, and 1 mM DTT.

Human wild-type full-length APTX gene was obtained from GenScript and cloned into pET-24b expression vector (Novagen). The recombinant his-tag protein was overexpressed in BL21(DE3) *E. coli* cells (Invitrogen) in LB media at 37 °C for 8 h and induced with 0.5 mM IPTG. The cells were harvested, lysed at 4 °C, and then clarified as described above. The supernatant was loaded onto a HisTrap HP column (GE Health Sciences) and purified with an increasing imidazole gradient (0–300 mM) elution at 4 °C. The collected fractions were then further purified by Superdex 200 Increase 10/300 chromatography (GE Healthcare) in the buffer containing 50 mM Tris-HCl (pH 7.5), 200 mM NaCl, and 1 mM DTT.

All proteins used in this study were dialyzed against the storage buffer containing 25 mM TrisHCl (pH 7.4), 100 mM KCl, 1 mM TCEP, and 10% glycerol, concentrated, frozen in liquid nitrogen, and stored at −80 °C in aliquots.

### Surface plasmon resonance assay for protein–protein interaction measurements

We analyzed the protein–protein interactions between XRCC1 (wild-type and disease-associated variants) and BER proteins (polβ, ligase IIIα, and APTX) by SPR in real time. The experiments were carried out using Biacore X-100 (GE Healthcare) at 25 °C. One flow cell of the CM5 sensor chip was activated with a 1:1 mixture of 0.2 M EDC and 0.05 M NHS in water, as described by the manufacturer, then the interacting protein partner of XRCC1 was injected over the flow cell in 10 mM sodium acetate at pH 4.0 (ligase IIIα), pH 5.0 (polβ), and pH 5.5 (APTX) at a flow rate of 10 μl/min. The binding sites were blocked using 1 M ethanolamine. XRCC1 wild-type or mutants ranging in the concentrations as indicated in the figures were then injected for 2 min (ligase IIIα) or 3 min (polβ and APTX) at a flow rate of 30 μl/min. The running buffer was the same as the protein storage buffer (20 mM HEPES pH 7.4, 150 mM NaCl, 3 mM EDTA and 0.005% (v/v) Surfactant P20). After a dissociation phase for 3 to 4 min, 10 mM Glycine-HCl (pH 2.0) was injected for 30 s to regenerate the chip surface. Nonspecific binding to a blank flow cell was subtracted to obtain corrected sensorgrams. All data were analyzed using BIAevaluation software version 2.0.1 and fitted to a 1:1 (Langmuir) binding model to obtain equilibrium constants (K*D*) for XRCC1 protein–protein interactions with polβ, ligase IIIα, and APTX.

### BioLayer interferometry assays for DNA-binding measurements

We analyzed DNA-binding kinetics of XRCC1 wild-type and disease-associated variants by BioLayer Interferometry (BLI) assays in real time. The binding kinetics for gap and nick DNA binding were performed using the Octet QKe system (Fortebio). BLI experiments were performed at 20 °C in 96-well microplates with agitation set to 1000 rpm. Oligodeoxyribonucleotides with and without a 3′-biotin label were obtained from Integrated DNA Technologies and used to prepare the one nucleotide gap DNA with template base C and the nick DNA including preinserted 3′-dG:C or 3′-8-oxodG:A ends (Table S2). Streptavidin (SA) biosensors (Fortebio) were used to attach the biotin-labeled DNA. SA biosensors were hydrated in the kinetics buffer containing 20 mM HEPES (pH 7.4), 200 mM NaCl, 0.5% BSA, and 0.05% Tween 20 at 20 °C for 20 min. The sensors were then immersed in DNA (40 nM) in the kinetics buffer for 300 s. After recording an initial baseline in the kinetics buffer (60 s), the sensors with DNA were exposed to the concentration range of XRCC1 or polβ for gap DNA binding and XRCC1 or ligase IIIα for nick DNA binding at the concentration range as indicated in the figures for 240 s association and then in kinetics buffer for 240 s dissociation. For gap DNA-binding measurements of polβ/XRCC1 complex in the presence of dGTP, the sensors with DNA were immersed in the reaction buffer containing 20 mM HEPES (pH 7.4), 200 mM NaCl, 0.5% BSA, 0.05% Tween 20, 10 mM MgCl_2_, and 0.1 mM dGTP for 120 s as the initial baseline, then exposed to the concentration range of polβ or preincubated mixture of polβ/XRCC1 (1:1) in the same reaction buffer for 240 s association and dissociation. Similarly, for nick DNA-binding measurements of ligase IIIα/XRCC1 complex in the presence of ATP, the sensors with DNA were immersed in the reaction buffer containing 20 mM HEPES (pH 7.4), 200 mM NaCl, 0.5% BSA, 0.05% Tween 20, 10 mM MgCl2, and 1 mM ATP for 120 s as the initial baseline, then exposed to the concentration range of ligase IIIα or preincubated mixture of ligase IIIα/XRCC1 proteins (1:1) in the same reaction buffer for 240 s association and dissociation. In all measurements, the affinity constants (K*D*), the association (k_on_) and dissociation (k_off_) rates were calculated using the ForteBio Data Analysis software with 1:1 binding model. The association rate = k_on_ [ligand][analyte] and the dissociation rate = k_off_ [ligand-analyte]. At equilibrium, forward and reverse rates are equal. All images were drawn using Graph Pad Prism 5.

### GST-pull-down assays

The GST-pull-down assays were performed to validate the protein–protein binding characteristics of polβ and XRCC1. Briefly, his-tagged XRCC1 proteins (5 μM) were incubated with GST-tag C-terminal domain of polβ (5 μM) in the assay buffer containing 50 mM Tris-HCl (pH 7.5), 100 mM NaCl, and 1 mM DTT at 4 °C for 2 h. The proteins were then mixed with 20 μl of glutathione sepharose beads (GE Healthcare) with constant rotation at 4 °C for 2 h. The beads were washed three times with the assay buffer and then by the elution buffer containing 50 mM Tris-HCl (pH 8.0) and 10 mM reduced glutathione. The eluted protein samples were analyzed on 12% SDS-PAGE, and the gels were scanned by AI680 (Amersham RGB).

### Size-exclusion chromatography of polβ/XRCC1 protein complexes

The protein complex of polβ and XRCC1 were obtained using SEC. Briefly, polβ (5 μM) and XRCC1 (5 μM) were prepared at equimolar 1:1 ratio of both proteins in the buffer containing 50 mM Tris (pH 8.0), 200 mM NaCl, and 1 mM DTT. The protein complexes were incubated for 2 h on ice prior to SEC analysis. SEC was performed using Superdex 200 increase GL 10/30 column (GE Healthcare) in the same buffer in which complexes were made. The fractions corresponding to the peaks were collected and analyzed for shifts in elution volumes for individual protein (polβ or XRCC1) *versus* protein complex (polβ/XRCC1). The fractions corresponding to the elution peaks were collected and analyzed on 12% SDS-PAGE, and the gels were scanned by AI680 (Amersham RGB).

### Polβ insertion coupled to ligation assay in the absence and presence of XRCC1

The one nucleotide gap DNA substrates with template A or C were used (Table S3) to test the ligation of polβ dGTP or 8-oxodGTP insertion products *in vitro* in the reaction mixture including polβ and ligase IIIα in the absence and presence of XRCC1 wild-type or mutants (V86R, R109A, P161L, R194W, R280H, R399Q, K431N, or Y576S). These BER assays were performed as described previously (67, 92, 93, 94, 95). Briefly, the reaction mixture (final volume of 10 μl) contains 50 mM Tris-HCl (pH 7.5), 100 mM KCl, 10 mM MgCl_2_, 1 mM ATP, 1 mM DTT, 100 μg ml^−1^ BSA, 10% glycerol, dGTP or 8-oxodGTP (100 μM), and DNA substrate (500 nM). The reaction was initiated by the addition of the polβ/XRCC1 protein complex (10 nM) into the reaction mixture containing DNA ligase IIIα (10 nM). The reaction mixtures were incubated at 37 °C for the time points as indicated in the figure legends. The reaction products were then mixed with an equal amount of gel loading buffer (95% formamide, 20 mM EDTA, 0.02% bromophenol blue, and 0.02% xylene cyanol) and separated by electrophoresis on an 18% polyacrylamide gel. The gels were finally scanned with a Typhoon PhosphorImager (Amersham Typhoon RGB), and the data were analyzed using ImageQuant software. BER reactions were performed similarly for the truncated proteins XRCC1▵BRCT-II and ligase IIIα▵BRCT.

### Ligation assays in the absence and presence of XRCC1

The nick DNA substrate with preinserted 3′-dG:C (Table S3) was used to test the nick sealing *in vitro* in a ligation assay including ligase IIIα in the absence and presence of XRCC1 wild-type or mutants (P161L, R194W, R280H, R399Q, K431N, or Y576S). Ligation assays were performed as described previously (67, 92, 93, 94, 95). Briefly, the reaction mixture (final volume of 10 μl) contains 50 mM Tris-HCl (pH 7.5), 100 mM KCl, 10 mM MgCl_2_, 1 mM ATP, 1 mM DTT, 100 μg ml^−1^ BSA, 10% glycerol, and DNA substrate (500 nM). The reaction was initiated by the addition of ligase IIIα (10 nM) alone or after its preincubation with XRCC1 (10 nM). The reaction mixtures were incubated at 37 °C for the time points as indicated in the figure legends. The reaction products were then mixed with an equal amount of gel loading buffer, separated by electrophoresis on an 18% polyacrylamide gel, and the data were analyzed using ImageQuant software as described above. The ligation reactions were performed similarly for the truncated proteins XRCC1▵BRCT-II and ligase IIIα▵BRCT.

## Data availability

All data are contained within the article. Further information and requests of materials used in this research should be directed to Melike Çaglayan (caglayanm@ufl.edu). Plasmid DNA constructs generated in this study will be made available *via* material transfer agreement (MTA).

## Supporting information

This article contains supporting information.

## Conflict of interest

The authors declare that they have no conflicts of interest with the contents of this article.

## Supporting information

Supplemental Figures S1–S16, Tables S1–S4 and Scheme S1
