# Supplementary material for: The scaffold protein XRCC1 stabilizes the formation of polβ/gap DNA and ligase IIIα/nick DNA complexes in base excision repair
Source: J Biol Chem. 2021 Jul 30;297(3):101025. doi: 10.1016/j.jbc.2021.101025 (PMC8405949; doi:10.1016/j.jbc.2021.101025)
Supplement: Supplemental Figures S1–S16, Tables S1–S4 and Scheme S1 [file mmc1.pdf]

SUPPLEMENTARY DATA

The scaffold protein XRCC1 stabilizes the formation of polβ/gap DNA and ligase IIIα/nicked DNA complexes in base excision repair

Qun Tang and Melike Çağlayan

Running title: XRCC1 orchestrating the downstream steps of coordinated BER

Department of Biochemistry and Molecular Biology, University of Florida, Gainesville, FL 32610, USA

To whom correspondence should be addressed. Tel.: +1 352-294-8383; Email: caglayanm@ufl.edu

Supplementary Scheme 1

Supplementary Figures 1-16

Supplementary Tables 1-4

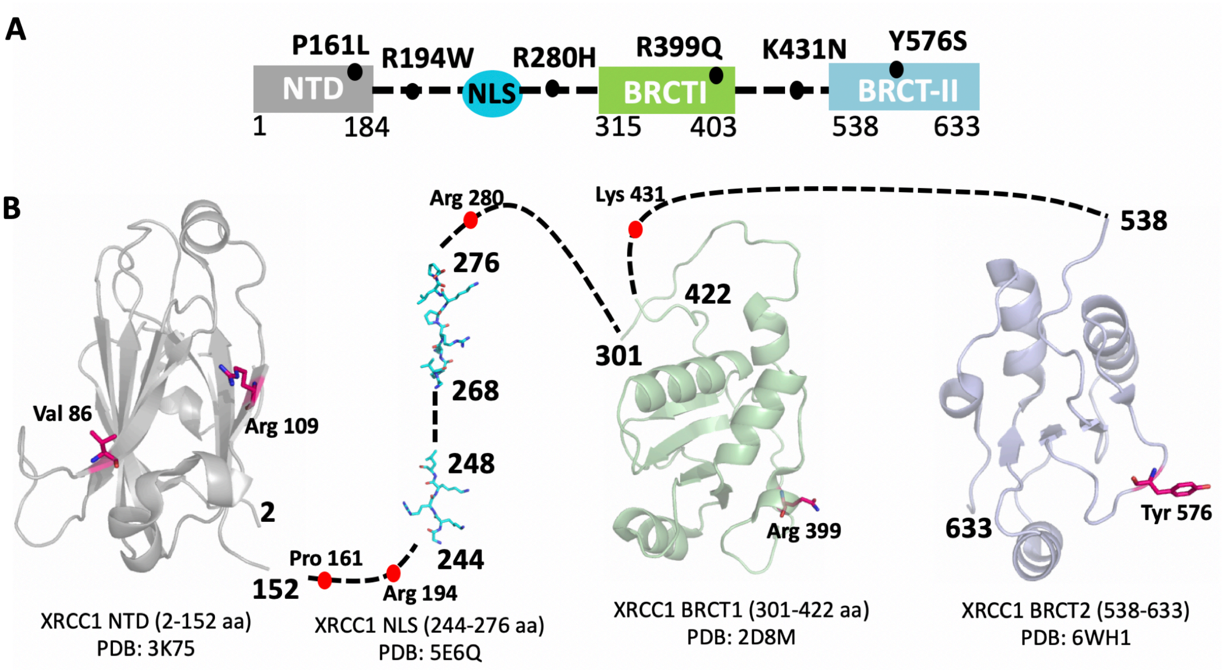

Supplementary Scheme 1. Protein domain organization of XRCC1 and its disease-associated variants used in this study. *A,* XRCC1 protein (1-633 aa) contains the N-terminal domain (NTD), nuclear localization signal (NLS), central BRCA1 C-terminal (BRCT) I domain and C-terminal BRCT-II domain. *B,* The mutations associated with SNP variants on the *XRCC1* gene (P161L, R194W, R280H, R399Q, Y576S, and K431N) are located on the different domains of XRCC1 protein as shown in structure models (ribbon diagram) with amino acid residues as sticks.

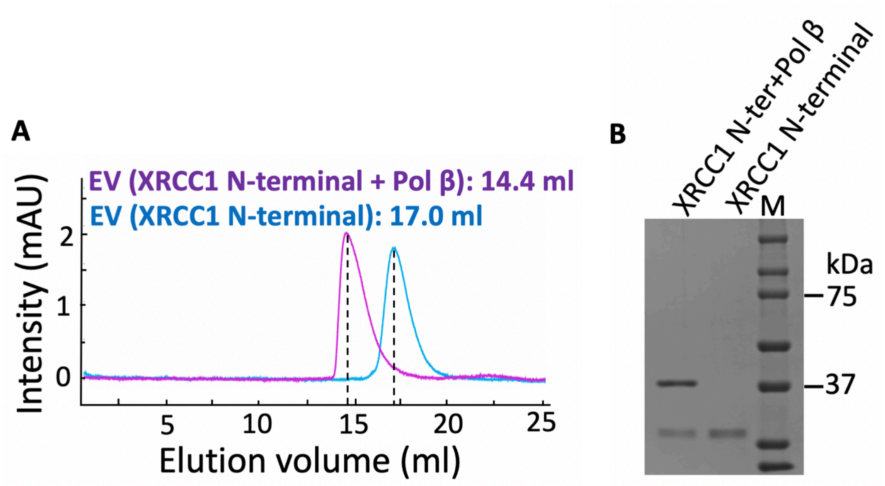

Supplementary Figure 1. Protein complex formation between XRCC1 N-terminal domain and polβ. *A,* Size-exclusion chromatography profiles of individual XRCC1 NTD protein and protein complex of XRCC1 NTD/polβ are presented as elution volumes (EV) in 17.0 and 14.4 ml, respectively. *B,* Each peak fraction is analyzed on 12% SDS-polyacrylamide (w/v) gel and compared with the molecular weight marker (M: Precision Plus Protein Dual Color Standards, 10-250 kDa).

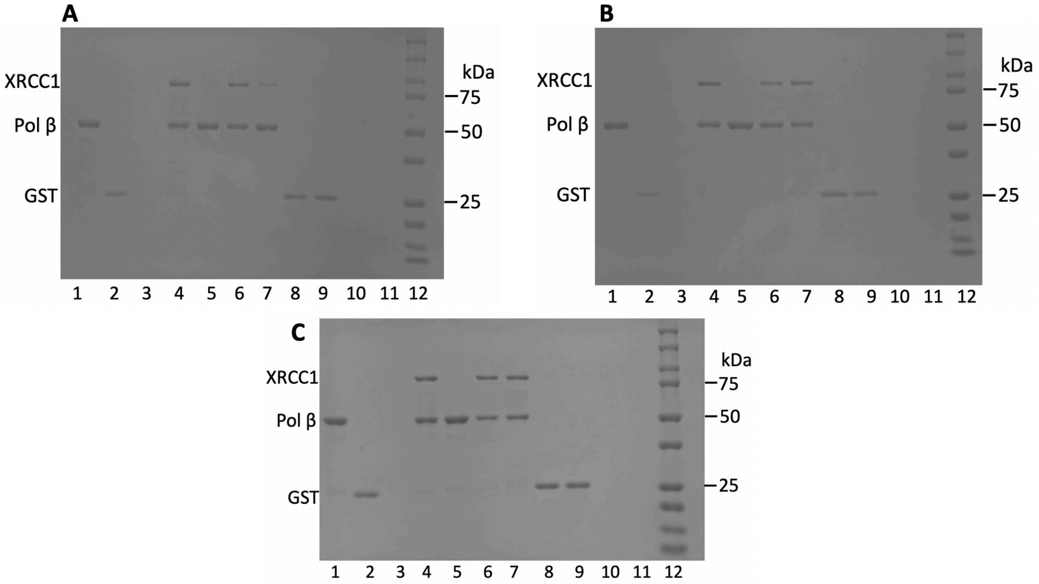

Supplementary Figure 2. GST-pull down analyses of XRCC1 and polβ interaction. Protein-protein binding interactions were performed using C-terminal domain of GST-tag polβ and full-length of his-tag XRCC1 (wild-type or mutant) by GST-pull down and analyzed on 10% SDS–polyacrylamide gels. *A-C,* Lanes 1-5 are polβ C-terminal, GST-tag alone, XRCC1 wild-type alone, XRCC1/polβ, and XRCC1 V86R/polβ, respectively. Line 12 is a protein marker (M: Precision Plus Protein Dual Color Standards, 10-250 kDa) *A,* Lanes 6-11 are P161L/polβ, R109A/polβ, P161L/GST, R109A/GST, P161L alone, and R109A alone, respectively. *B,* Lanes 6-11 are R399Q/polβ, R280H/polβ, R399Q/GST, R280H/GST, R399Q alone, and R280H alone, respectively. *C,* Lanes 6-11 are R194W/polβ, Y576S/polβ, R194W/GST, Y576S/GST, R194W alone, and Y576S alone, respectively.

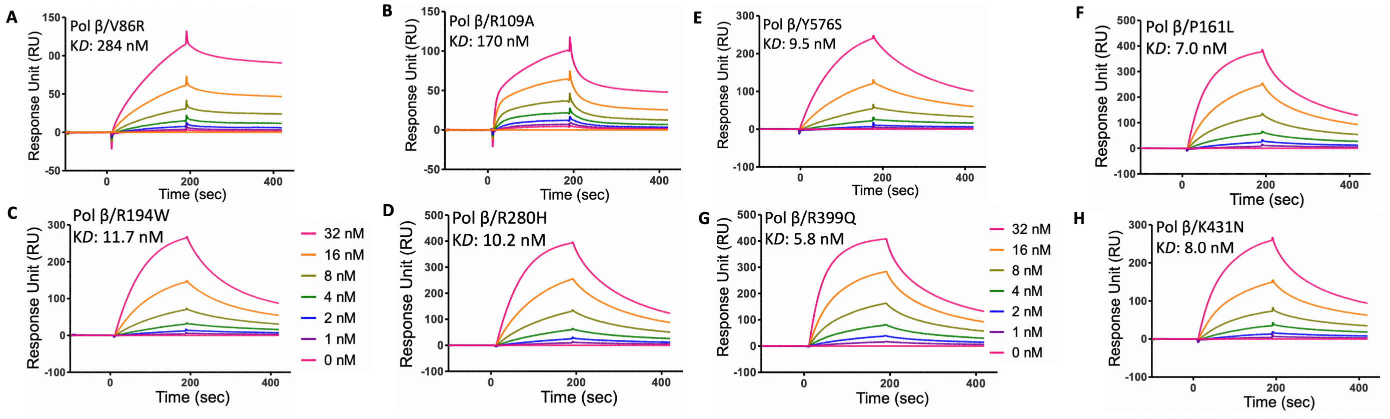

Supplementary Figure 3. Protein-protein interaction analyses between polβ and XRCC1 variants. The real-time protein-protein interaction measurements between polβ and XRCC1 interaction mutants V86R (*A*), R109A (*B*), and XRCC1 variants R194W (*C*), R280H (*D*), Y576S (*E*), P161L (*F*), R399Q (*G*), and K431N (*H*) were performed by SPR assay where polβ was immobilized on CM5 biosensors. The ligand association and dissociation phases are shown for the protein concentrations range of XRCC1 (0-32 nM) on the side of sensorgrams. The comparison table showing the differences in the equilibrium binding constants (K*D*) of XRCC1 wild-type and variants is presented in Fig. 2*D*.

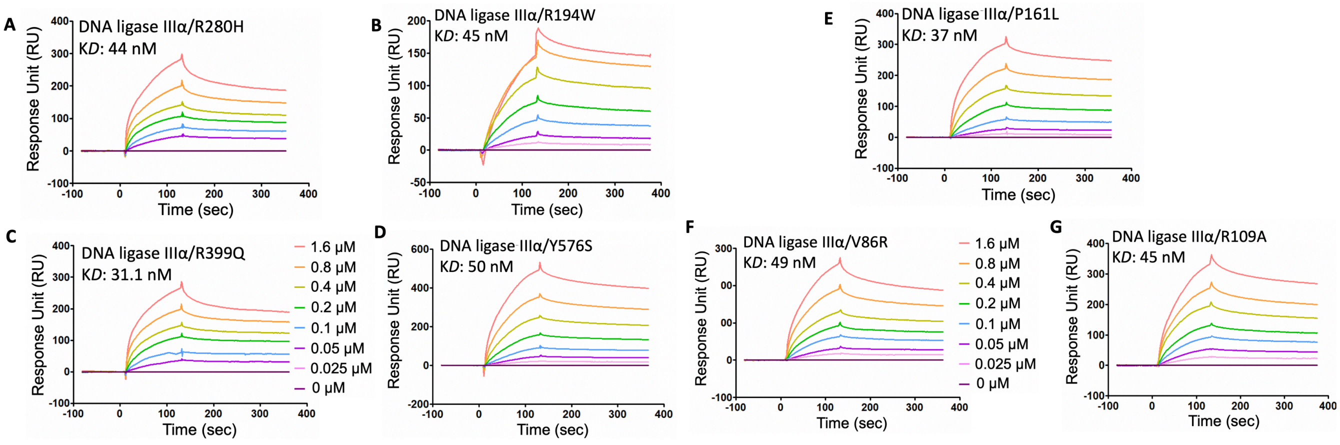

Supplementary Figure 4. Protein-protein interaction analyses between DNA ligase IIIα and XRCC1 variants. The real-time protein-protein interaction measurements between DNA ligase IIIα and XRCC1 variants R280H (*A*), R194W (*B*), R399Q (*C*), Y576S (*D*), P161L (*E*) and polβ/XRCC1 interaction mutants V86R (*F*), R109A (*G*) were performed by SPR assay where ligase IIIα was immobilized on CM5 biosensors. The ligand association and dissociation phases are shown for the protein concentrations range of XRCC1 (0-1.6 μM) on the side of sensorgrams. The comparison table showing the differences in the equilibrium binding constants (K*D*) of XRCC1 wild-type and variants is presented in Fig. 2*D*.

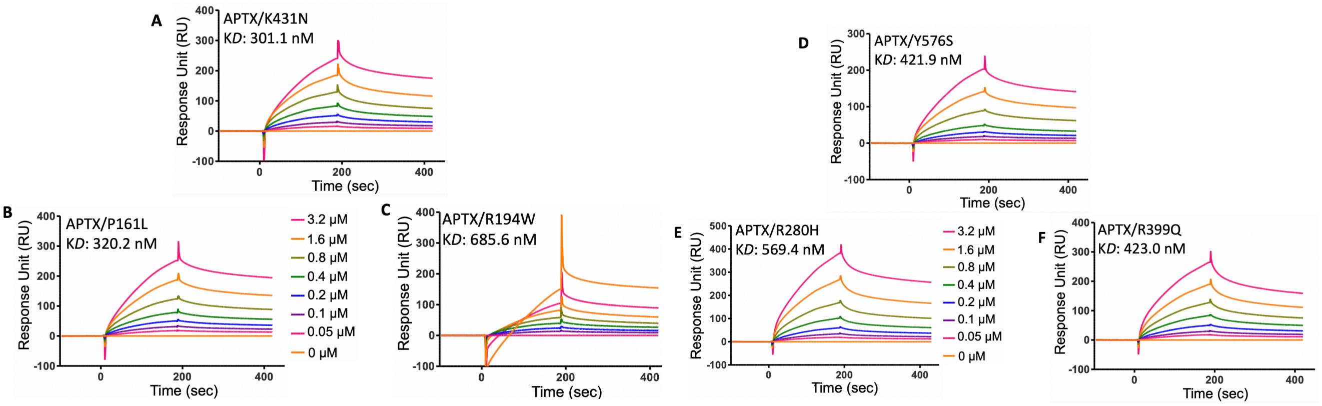

Supplementary Figure 5. Protein-protein interaction analyses between APTX and XRCC1 variants. The real-time protein-protein interaction measurements between APTX and XRCC1 variants K431N (*A*), P161L (*B*), R194W (*C*), Y576S (*D*), R280H (*E*) and R399Q (*F*) were performed by SPR assay where APTX was immobilized on CM5 biosensors. The ligand association and dissociation phases are shown for the protein concentrations range of XRCC1 (0-3.2 μM) on the side of sensorgrams. The comparison table showing the differences in the equilibrium binding constants (K*D*) of XRCC1 wild-type and variants is presented in Fig. 2*D*.

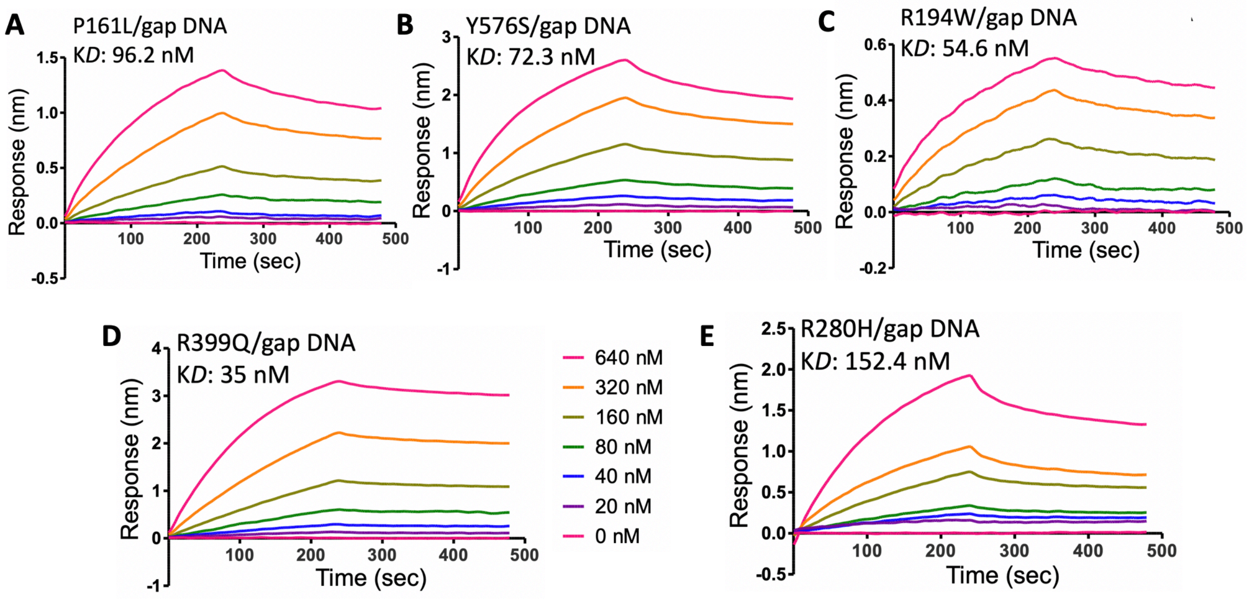

Supplementary Figure 6. One nucleotide gap DNA binding kinetics of XRCC1 variants. The real-time DNA binding kinetics of XRCC1 variants P161L (*A*), Y576S (*B*), R194W (*C*), R399Q (*D*) and R280H (*E*) were performed by biolayer interferometry analyses for the concentrations range of XRCC1 (0-640 nM). The comparison table showing the differences in the equilibrium binding constants (K*D*) of XRCC1 wild-type and cancer-associated variants is presented in Fig. 3*D*.

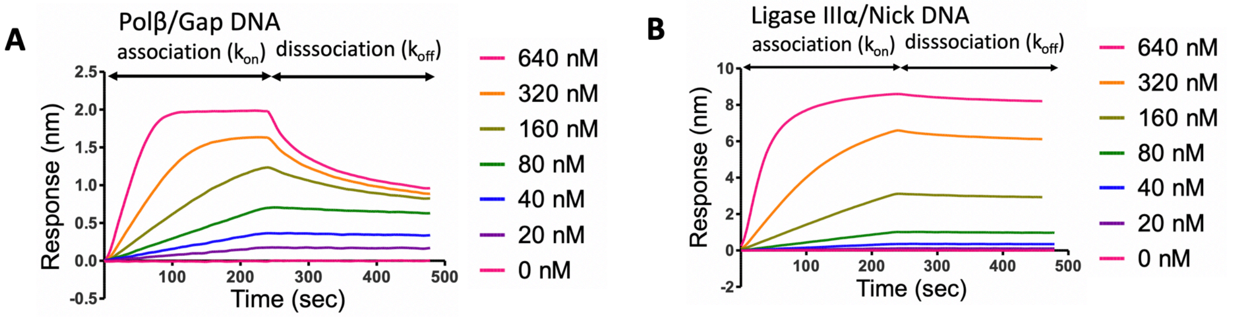

C

Gap DNA	*KD* (nM)	k_{on} (10^4)	k_{off} (10^{-3})
XRCC1	110.0	1.0	1.1
Polβ	5.44	51	2.7

D

Nick DNA	*KD* (nM)	k_{on} (10^4)	k_{off} (10^{-3})
XRCC1	54.0	1.4	0.8
Ligase IIIα	4.9	4.7	23

Supplementary Figure 7. Gap and nick DNA binding kinetics of polβ and DNA ligase IIIα. The real-time DNA binding kinetics of polβ (*A*) for gap DNA and ligase IIIα (*B*) for nick DNA. *C-D*, Tables show the comparison for the equilibrium binding constants (K*D*), the association (k_{on}) and dissociation (k_{off}) rates between polβ, ligase IIIα, and XRCC1.

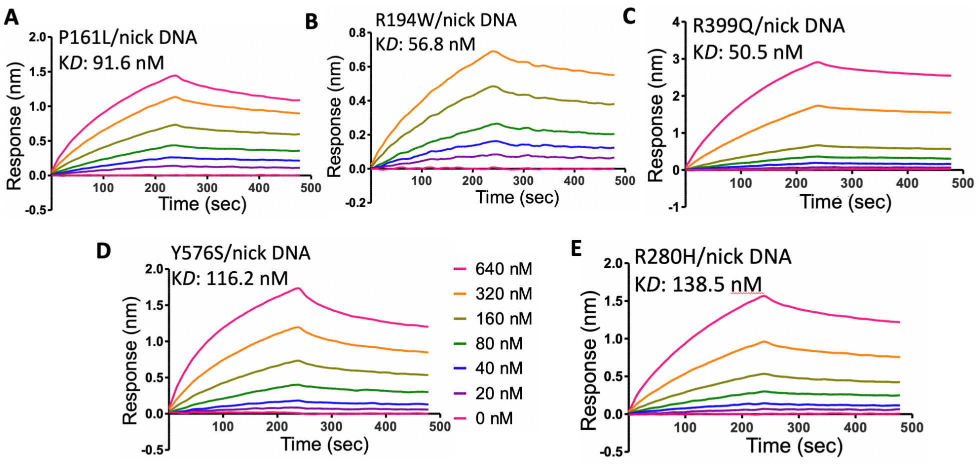

Supplementary Figure 8. DNA binding kinetics of XRCC1 variants to nick repair intermediate with preinserted 3'-dG:C. Nick DNA binding measurements for XRCC1 variants P161L (*A*), R194W (*B*), R399Q (*C*), Y576S (*D*), and R280H (*E*) were performed by biolayer interferometry analyses for the concentrations range of XRCC1 (0-640 nM). The comparison table showing the differences in the equilibrium binding constants (K*D*) of XRCC1 wild-type and cancer-associated variants is presented in Fig. 3*D*.

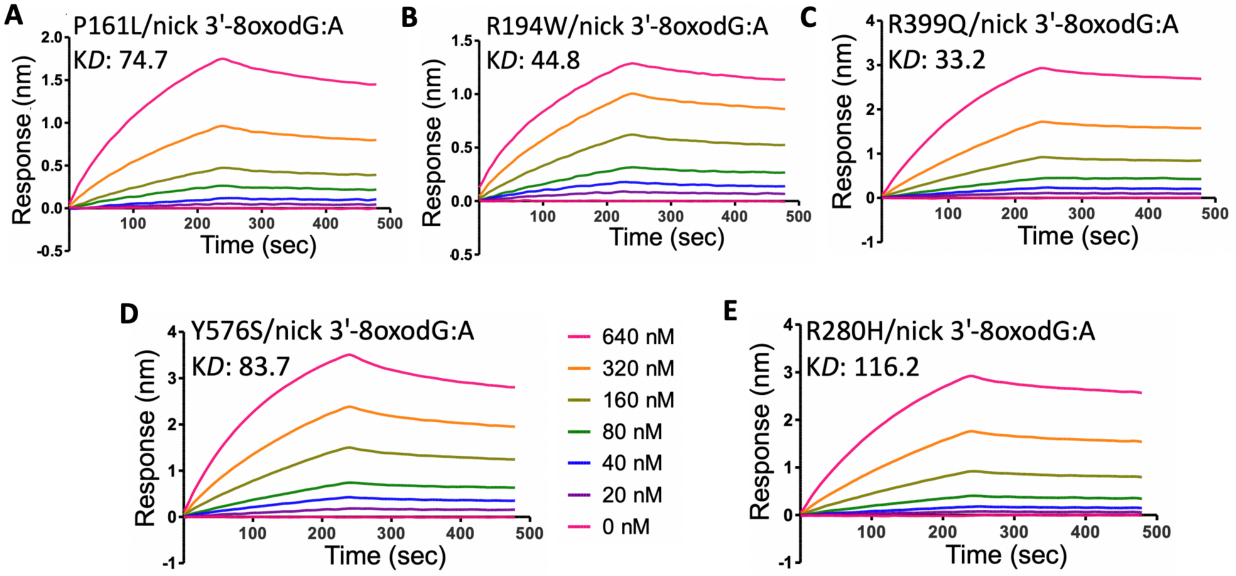

Supplementary Figure 9. DNA binding kinetics of XRCC1 variants to nick repair intermediate with preinserted 3'-8-oxodG:A. Nick DNA binding measurements for XRCC1 variants P161L (*A*), R194W (*B*), R399Q (*C*), Y576S (*D*), and R280H (*E*) were performed by biolayer interferometry analyses for the concentrations range of XRCC1 (0-640 nM). The comparison table showing the differences in the equilibrium binding constants (K*D*) of XRCC1 wild-type and cancer-associated variants is presented in Fig. 3*D*.

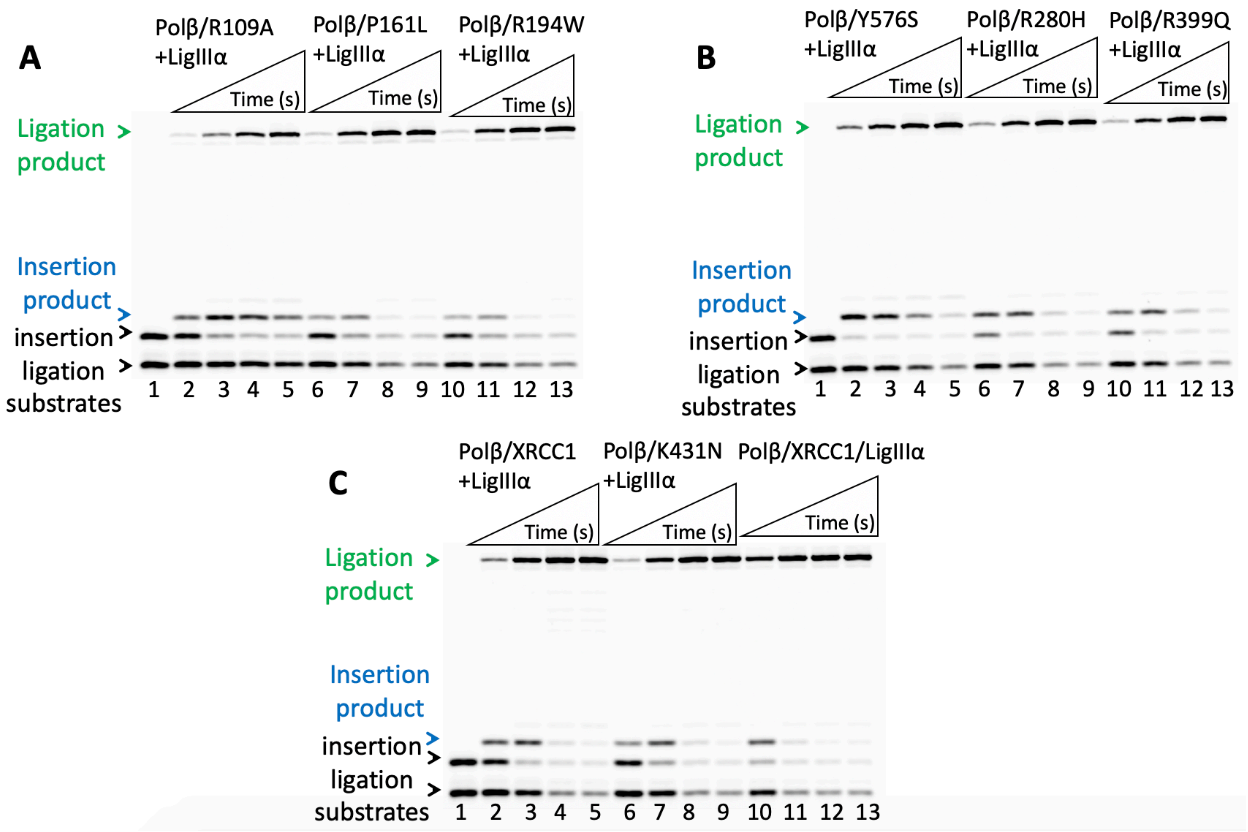

Supplementary Figure 10. Ligation of polβ dGTP insertion products by ligase IIIα in the presence of XRCC1 variants. *A-C,* Line 1 is the negative enzyme control of the one nucleotide gap DNA substrate. *A,* Lanes 2-5, 6-9, and 10-13 are polβ dGTP:C insertion coupled to ligation products in the presence of XRCC1 variants R109A, P161L, and R194W, respectively, and correspond to time points of 10, 30, 45, and 60 sec. *B,* Lanes 2-5, 6-9, and 10-13 are polβ dGTP:C insertion coupled to ligation products in the presence of XRCC1 variants Y576S, R280H, and R399Q, respectively, and correspond to time points of 10, 30, 45, and 60 sec. *C,* Lanes 2-5, 6-9, and 10-13 are polβ dGTP:C insertion coupled to ligation products in the presence of XRCC1 wild-type, K431N, and the protein complex of polβ/XRCC1/ligase IIIα, respectively, and correspond to time points of 10, 30, 45, and 60 sec. The bar graphs showing the comparisons in the amount of ligation products between XRCC1 wild-type and variants are presented in Fig. 4.

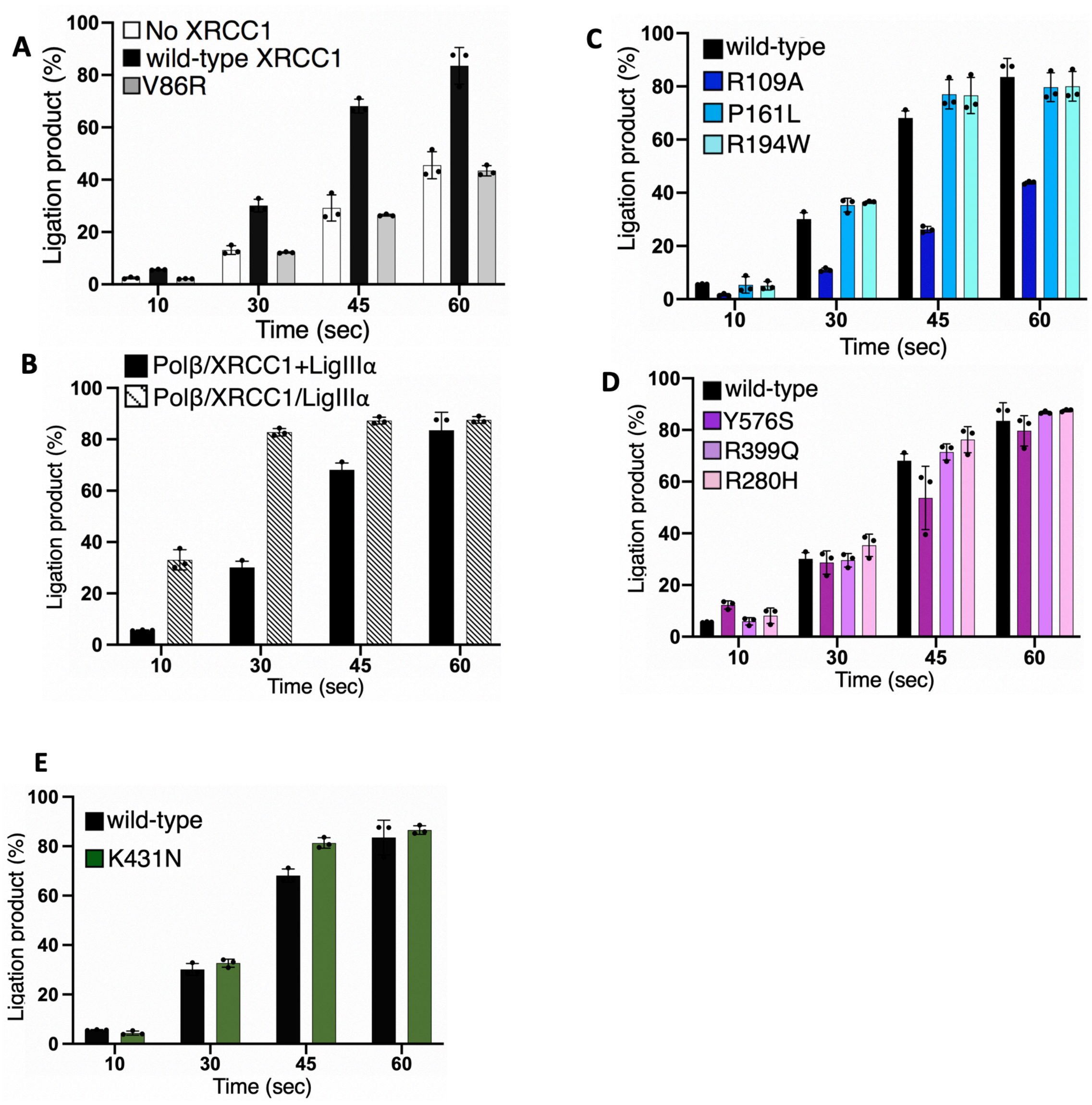

Supplementary Figure 11. The bar graphs with individual data points for three repeat experiments show time-dependent changes in the amount of ligation products in the absence or presence of XRCC1 (*A*), polβ/XRCC1/ligase IIIα complex (*B*), and XRCC1 disease-associated variants (*C*-*E*). The data represent the average of three independent experiments ± SD. The gel images are presented in Fig. 4 and S10.

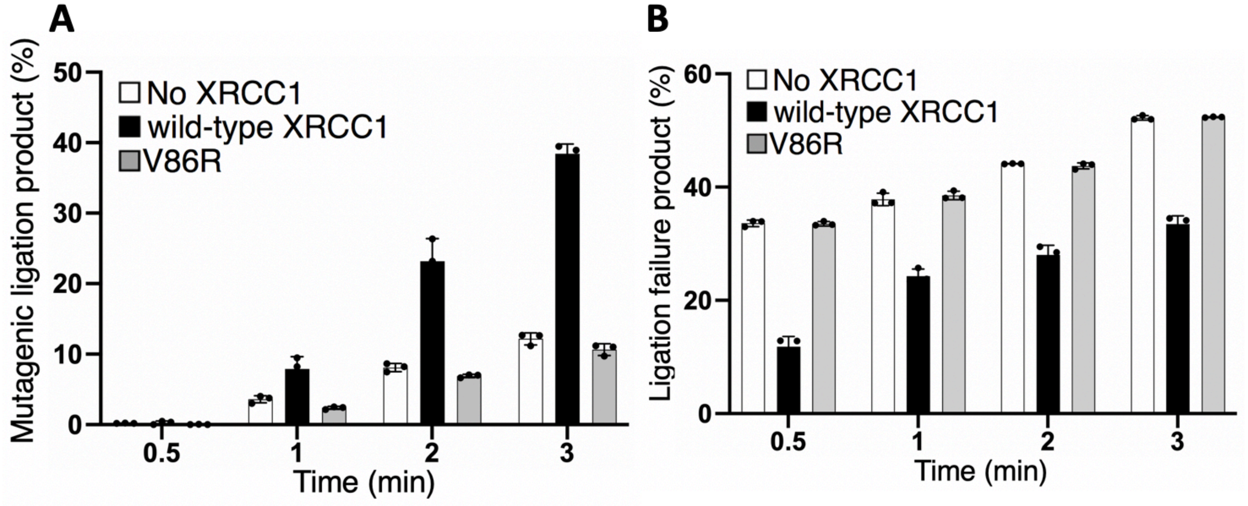

Supplementary Figure 12. Ligation of polβ 8-oxodGTP insertion products by ligase IIIα in the presence and absence of XRCC1. The bar graphs with individual data points for three repeat experiments show time-dependent changes in the amount of mutagenic ligation (*A*) and ligation failure (*B*) products. The data represent the average of three independent experiments ± SD. The gel images are presented in Fig. 6.

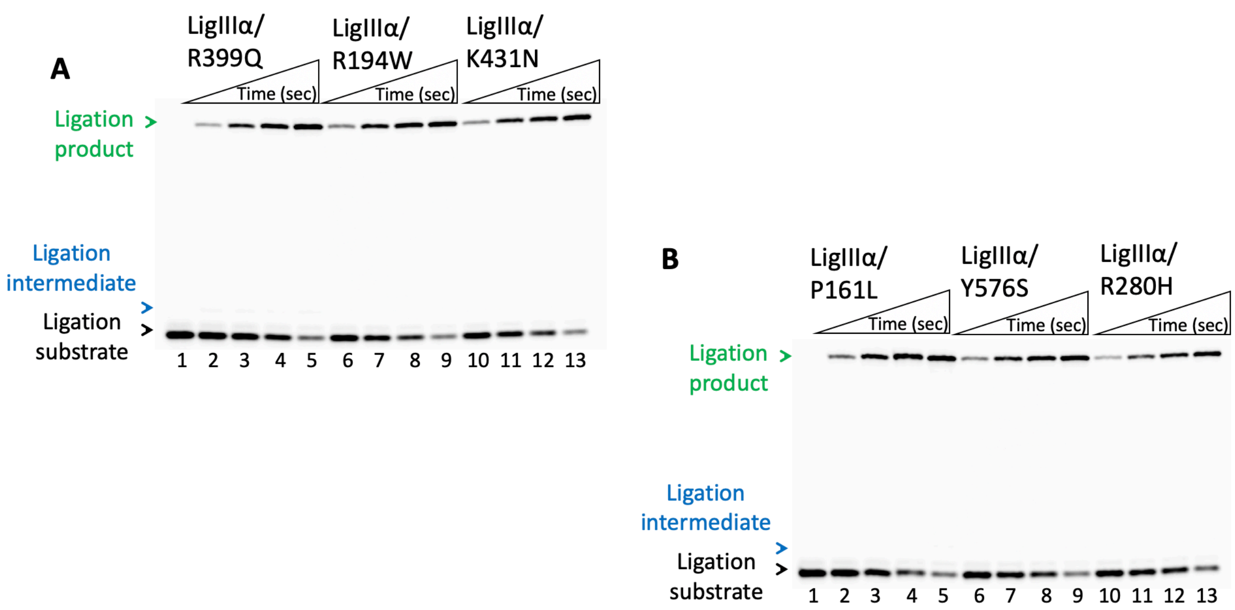

Supplementary Figure 13. Ligation of nick DNA by ligase IIIα in the presence of XRCC1 variants. *A,* Line 1 is the negative enzyme control of the nick DNA substrate. Lanes 2-5, 6-9, and 10-13 are the ligation products in the presence of XRCC1 variants R399Q, R194W, and K431N, respectively, and correspond to time points of 10, 20, 30, and 40 sec. *B,* Line 1 is the negative enzyme control of the nick DNA substrate. Lanes 2-5, 6-9, and 10-13 are the ligation products in the presence of XRCC1 variants P161L, Y576S, and R280H, respectively, and correspond to time points of 10, 20, 30, and 40 sec. The bar graphs showing the comparisons in the amount of ligation products between XRCC1 wild-type and variants are presented in Fig. 8.

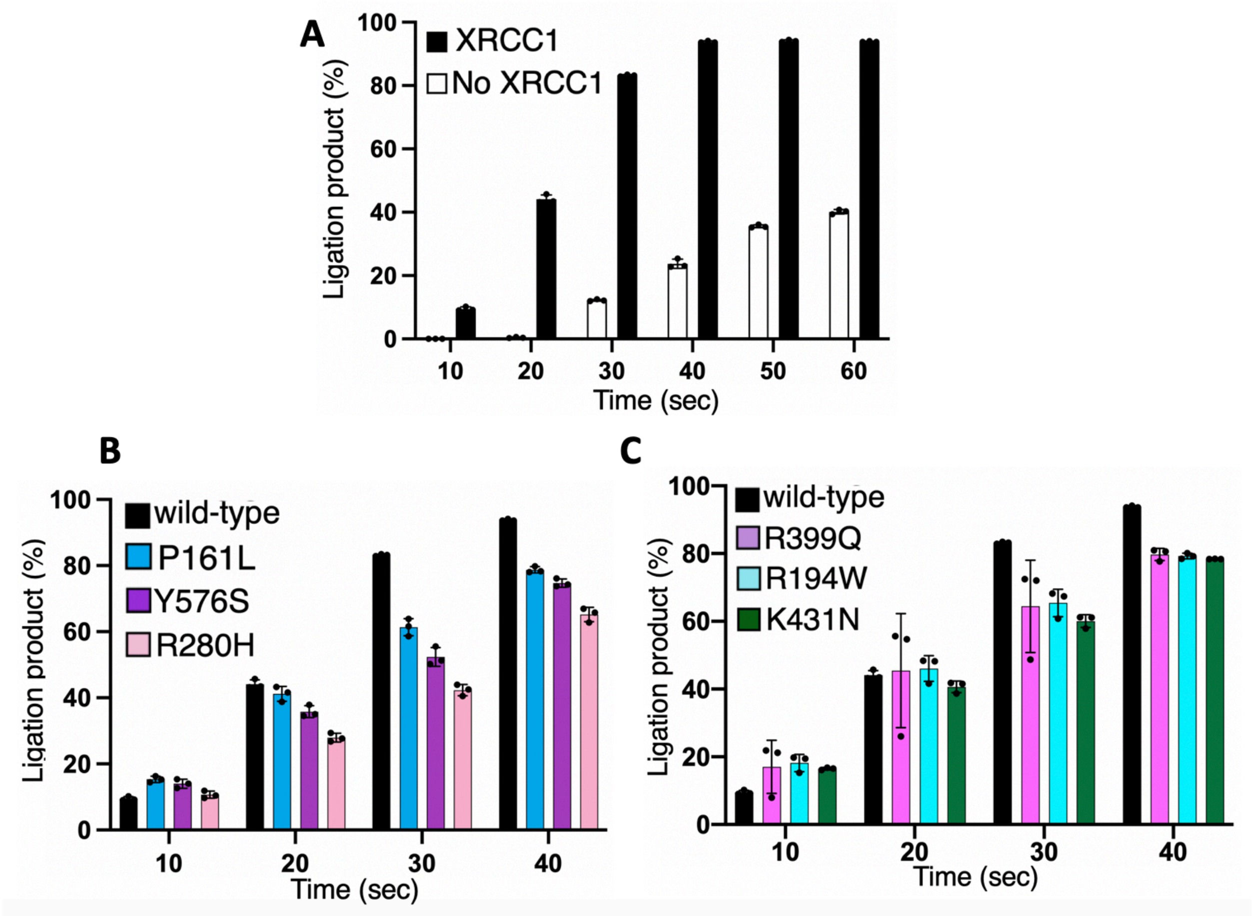

Supplementary Figure 14. The bar graphs with individual data points for three repeat experiments show time-dependent changes in the amount of ligation products in the absence or presence of XRCC1 (*A*) and XRCC1 disease-associated variants (*B-C*). The data represent the average of three independent experiments ± SD. The gel images for XRCC1 wild-type and variants are presented in Fig. 8 and S13.

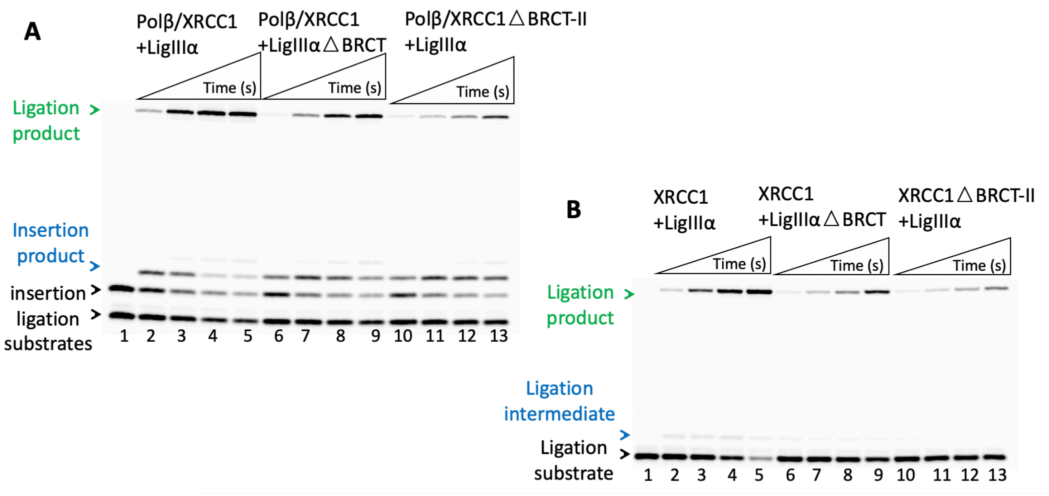

Supplementary Figure 15. Impact of BRCT domains on the downstream steps of BER pathway. *A,* Impact of BRCT domain on the ligation of polβ dGTP insertion products. Line 1 is the negative enzyme control of the one nucleotide gap DNA substrate. Lanes 2-5, 6-9, and 10-13 are the ligation of polβ dGTP:C insertion products in the presence of XRCC1/ligase IIIα, XRCC1/ligase IIIαΔBRCT, XRCC1ΔBRCT-II/ligase IIIα, respectively, and correspond to time points of 10, 30, 45, and 60 sec. *B,* Impact of BRCT domain on the ligation of nick repair intermediate. Line 1 is the negative enzyme control of the nick DNA substrate. Lanes 2-5, 6-9, and 10-13 are the ligation products in the presence of XRCC1/ligase IIIα, XRCC1/ligase IIIαΔBRCT, XRCC1ΔBRCT-II/ligase IIIα, respectively, and correspond to time points of 10, 20, 30, and 40 sec. The graphs showing time-dependent changes in the amount of ligation products are presented in Fig. 10.

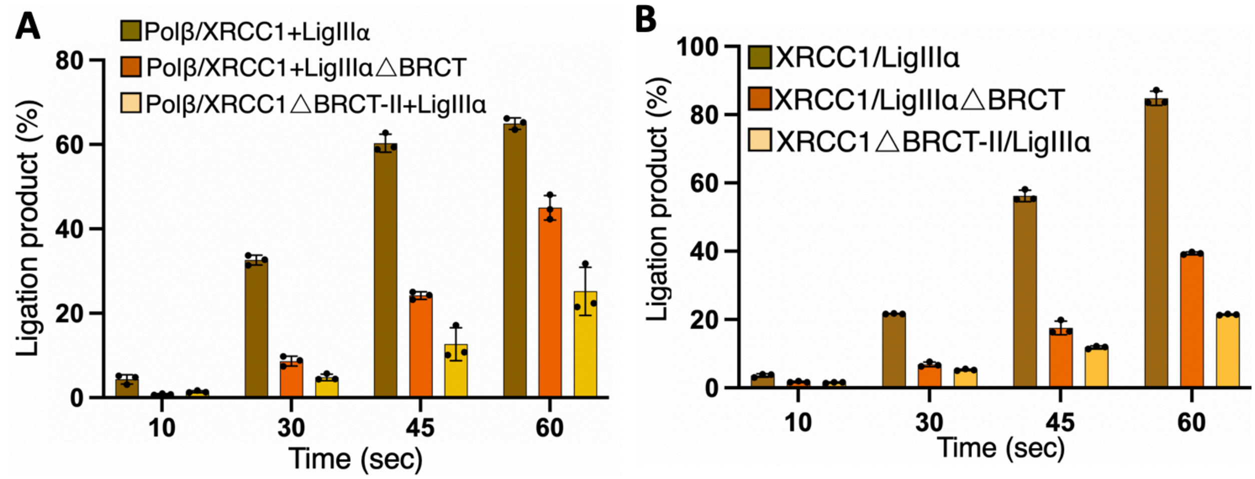

Supplementary Figure 16. Impact of XRCC1/ligase IIIα interacting BRCT domains on the ligation of polβ nucleotide insertion products and nick sealing. The bar graphs with individual data points for three repeat experiments show time-dependent changes in the amount of ligation products for ligation of polβ dGTP insertion products (*A*) and nicked repair intermediate (*B*) . The data represent the average of three independent experiments ± SD. The gel images are presented in Fig. 10 and S15.

Primer	Sequence (5'-3')
Wild-type full-length XRCC1-F	CGGTCATATGATGCCGGAGATCCGCC
Wild-type full-length XRCC1-R	CGAGTCGACGGCTTGCGGCACCACC
Wild-type N-terminal XRCC1-F	CGGTCATATGATGCCGGAGATCCGCC
Wild-type N-terminal XRCC1-R	CTGGTCGACAGAGTTGGCGCTCTCATCCT
V86R-F	ATGAGGTGACCAGAAGGCGCTCATAGTCTTGCTCCC
V86R-R	GGGAGCAAGACTATGAGCGCCTTCTGGTCACCTCAT
R109A-F	CAGGCCCAAACATGGCAACGCGGTTGGGGT
R109A-R	ACCCCAACCGCGTTGCCATGTTTGGGCCTG
P161L-F	CACCTTCTGGGACAGGGCCTCTGCCTC
P161L-R	GAGGCAGAGGCCCTGTCCCAGAAGGTG
R194W-F	GGATGTCTTGTTGATCCAGCTGAAGAAGAGAGCCC
R194W-R	GGGCTCTCTTCTTCAGCTGGATCAACAAGACATCC
R280H-F	CTGTGGCTGGGGTATGAGTTGGAGCTGGC
R280H-R	GCCAGCTCCAACTCATACCCCAGCCACAG
R399Q-F	CATGAGGTACCTCTGGGAGGGCAGCCG
R399Q-R	CGGCTGCCCTCCCAGAGGTACCTCATG
Y576S-F	CCCGGTCACTCATAGAGTCCTCGAGCTCC
Y576S-R	GGAGCTCGAGGACTCTATGAGTGACCGGG

Supplementary Table 1. The oligonucleotide primers used for the cloning of XRCC1 wild-type (full-length and N-terminal), polβ interaction mutants V86R, R109A, and cancer-associated variants P161L, R194W, Y576S, R280H, and R399Q into pET-24b expression vector.

DNA substrates	Sequence
One nucleotide gap DNA with template base C	P<br>5'-CATGGGCGGCATGAACC GAGGCCCATCCTCACC-3'-Bio<br>3'-GTACCCGCCGTACTTGGCCTCCGGGTAGGAGTGG-5'
Nick DNA with 3'-dG:C	P<br>5'-CATGGGCGGCATGAACCGGAGGCCCATCCTCACC-3'-Bio<br>3'-GTACCCGCCGTACTTGGCCTCCGGGTAGGAGTGG-5'
Nick DNA with 3'-8-oxodG:A	P<br>5'-CATGGGCGGCATGAACCXGAGGCCCATCCTCACC-3'-Bio<br>3'-GTACCCGCCGTACTTGGACTCCGGGTAGGAGTGG-5'

Supplementary Table 2. One nucleotide gap and nicked DNA substrates used in BLI assays to analyze DNA binding affinity of XRCC1 wild-type and mutants. The positions of template bases are undelined. Bio stands for biotin-labeled at 3'-end of DNA substrates. X is for preinserted 3'-8-oxodG and P is phosphate group.

DNA substrates	Sequence
One nucleotide gap DNA with template base C	P<br>FAM-5'-CATGGGCGGCATGAACC GAGGCCCATCCTCACC-3'-FAM<br>3'-GTACCCGCCGTACTTGGCCTCCGGGTAGGAGTGG-5'
One nucleotide gap DNA with template base A	P<br>FAM-5'-CATGGGCGGCATGAACC GAGGCCCATCCTCACC-3'-FAM<br>3'-GTACCCGCCGTACTTGGACTCCGGGTAGGAGTGG-5'
Nick DNA with 3'-dG:C	P<br>5'-CATGGGCGGCATGAACCGGAGGCCCATCCTCACC-3'-FAM<br>3'-GTACCCGCCGTACTTGGCCTCCGGGTAGGAGTGG-5'

Supplementary Table 3. The one nucleotide gap and nick DNA substrates used in reconstituted repair assays (coupled and ligation, respectively) *in vitro*. FAM denotes a flourescence tag and is located at 5'- and/or 3'-ends of DNA substrates. The base at template base position is underlined and P is phosphate group.

A

XRCC1/Gap DNA	*KD* (nM)	k_{on} (10^4)	k_{off} (10^{-3})
wild type	110	1.0	1.1
P161L	96.2	1.3	1.3
R194W	54.6	1.9	0.8
R280H	152.4	1.2	1.8
R399Q	35	1.3	0.45
Y576S	72.3	1.8	1.3

B

XRCC1/Nick DNA with 3'-dG:C	*KD* (nM)	k_{on} (10^4)	k_{off} (10^{-3})
wild type	54.0	1.4	0.8
P161L	91.6	1.5	1.3
R194W	56.8	1.7	1.0
R280H	138.5	0.9	1.2
R399Q	50.5	1.2	0.6
Y576S	116.6	1.3	1.5

C

XRCC1/Nick DNA with 3'-8-oxodG:A	*KD* (nM)	k_{on} (10^4)	k_{off} (10^{-3})
wild type	75.4	1.3	1.0
P161L	74.7	1.2	0.8
R194W	44.8	1.4	0.6
R280H	116.2	0.9	1.0
R399Q	33.2	1.5	0.5
Y576S	83.7	1.6	1.3

Supplementary Table 4. **DNA binding kinetics of XRCC1 wild-type and cancer-associated variants.** One nucleotide gap (*A*) and nick DNA with preinserted 3'-dG:C (*B*) or 3'-8oxodG:A (*C*) binding kinetics of XRCC1 wild-type and variants. The real-time DNA binding measurements were performed by biolayer interferometry analyses and the equilibrium binding constants (K*D*), the association (k_{on}) and dissociation (k_{off}) rates were calculated using the ForteBio data analysis software with 1:1 binding model.
